# A protocol for a cluster randomized controlled trial to assess the impact of Balika Bodhu: A combined empowerment and social norm based sexual and reproductive health and rights intervention for married adolescent girls in rural Bangladesh

**DOI:** 10.1371/journal.pone.0304988

**Published:** 2024-08-23

**Authors:** Mahfuz Al Mamun, Sultan Mahmud, Jannatul Ferdous Antu, Aloka Talukder, Ruchira Tabassum Naved

**Affiliations:** Maternal and Child Health Division, icddr,b, Dhaka, Bangladesh; Independent Consultant, UNITED STATES

## Abstract

**Introduction:**

Sexual and reproductive health and rights (SRHR) are vital for both individual well-being and development. Bangladesh has made long strides in improving SRHR over the last few decades. However, the progress has been uneven across various groups of reproductive-aged females, with the married adolescent girls (MAGs) often being more vulnerable to denial of SRHR than other women. This study intends to develop Balika Bodhu, a combined empowerment and social norm intervention for promoting SRHR among the MAGs and assess its impact.

**Methods:**

The evaluation will employ a mixed-method two-arm Cluster Randomized Controlled Trial (CRCT) design, where Arm 1 receives the intervention, and Arm 2 serves as the control. The trial will cover 32 clusters (villages) in Rajbari Sadar sub-district, randomized into two equally distributed study arms. A total of 1,120 MAGs aged 15–19 years will be randomly selected from the clusters (35 per cluster) to form a cohort. The MAGs, their husbands, selected elderly women (26 per village) and influential community members (26 per village) will receive group sessions in the intervention clusters. The MAGs and their husbands will be interviewed at baseline and endline. A randomly selected cross-sectional sample of community members aged 35–59 years at baseline and endline will also be surveyed to measure attitudes and social norm regarding SRHR of MAGs. Qualitative data will be collected using 32 In-depth Interviews, six Key Informant Interviews, and eight Focus Group Discussions from two intervention villages. Intention-to-treat (ITT) analysis will be performed to assess the impact of the intervention. Narrative analysis and the Grounded Theory approach will be used to analyze the qualitative data.

**Conclusion:**

Rigorous evaluation of Balika Bodhu should contribute to the literature on what works and what does not in addressing denial of SRHR to MAGs using empowerment and social norm intervention and inform policies and programs.

**Trial registration:**

Clinicaltrials.gov: identifier: NCT06126770; Date: Oct 7, 2023. Version 1.

## Introduction

Sexual and reproductive health and rights (SRHR) are vital for both individual well-being and development. It is recognized as essential for achieving social justice [1]. Achieving sexual and reproductive health rests on realizing related rights, including the right to control one’s own body, define one’s sexuality, choose one’s partner, and receive confidential, respectful, and high-quality health services [2]. However, denial of SRHR has been pervasive in many countries, and particularly in countries with patriarchal social structures, where female power and status are compromised.

At this backdrop, Bangladesh, characterized by patriarchal social structures, has made remarkable progress in improving contraceptive use and maternal health over the last few decades [3, 4]. However, the progress has not been consistent in different SRHR domains or across different groups of reproductive aged females [5]. Thus, for instance, choice and consent of females are often ignored in all spheres of SRHR. Patriarchal gender and social norms, as well as discriminatory laws and practices in Bangladesh perpetuate denial of SRHR to females [6, 7].

Marriage is almost universal in Bangladesh [8]. High prevalence of child marriage here (59%) results in a huge proportion of married adolescent girls in the population [3]. Most marriages are arranged and village exogamy is largely practiced. Thus, upon entry into marriage most girls are cut off from their own networks. Both gender and age hierarchies are very strong in this country. So, these child brides typically command the lowest power in the marital home. They typically have lower education than those who get married in adulthood. They lack voice and bargaining skills [9, 10]. The husband and/or the mother-in-law figure features prominently in decisions about their contraceptive use and timing of childbearing [10–15]. Moreover, childbearing often becomes a way to improve one’s standing in the marital home [9, 12]. These girls end up having teenage pregnancy, shorter birth intervals, and higher number of children [12, 16, 17]. Denial of SRHR usually becomes part of their lived experiences.

Although social norm is recognized as one of the main drivers of denial of SRHR to married adolescent girls (MAGs) [18, 19], social norm theories and approaches to programming are relatively new and there has been little attempt to develop and rigorously test interventions to address social norms worldwide. In line with other proponents of social norm interventions, we argue that in contexts where norms are significant drivers of decision-making and behavior, social norm interventions are most likely to be effective in addressing the issues sustainably [20, 21]. A handful of social norm interventions have been developed for addressing adolescent SRHR (ASRHR) in low- and middle-income countries (LMIC). They are mainly focused on child marriage [22], violence [23] and female genital mutilation [24]. To date, a very few social norm interventions addressing other ASRHR issues (e.g., rights to define timing of conception, right to use contraceptives) have been rigorously evaluated. The two important qualitative evaluations in this area are CARE developed Tesfa [25] and Abdiboru [26] implemented in Africa. These projects aimed at promoting ASRHR knowledge, positive attitudes and uptake of SRH services empowering the MAGs and creating an enabling environment in the communities they live in. One of the most recent social norm interventions addressing ASRHR of married girls was the IMAGINE, another CARE developed project implemented in rural Bangladesh [27, 28]. Evaluation of these CARE projects seem to show some promise. However, none of them have been rigorously evaluated.

Over several decades many interventions aimed to promote SRHR through empowerment of partnered women and adolescents worldwide. We argue that conceptualization of these programs was often not grounded in solid theory and evidence. Thus, these programs often ignored some key components such as movement building, collective agency and critical consciousness, without which empowerment cannot take place in its true sense [29, 30]. Following feminist traditions, we define empowerment as intrinsic power within to overcome dominated consciousness, instrumental power to pursue aspirations, and collective power with others to pursue shared goals [31, 32]. We intend to address the above mentioned research and intervention gaps in the current study by developing and rigorously assessing the impact of Balika Bodhu, a combined social norm and comprehensive empowerment intervention to promote SRHR among the MAGs in rural Bangladesh.

### Objectives

The overall objective of this study is to develop and test Balika Bodhu, a combined empowerment and social norm intervention to address denial of sexual and reproductive health and rights to married adolescent girls. The specific objectives of this study are:

1. To develop Balika Bodhu to promote SRHR among the MAGs, particularly in the domains of contraception use, conception, and SRH service uptake.
2. To measure the effect of Balika Bodhu in increasing the MAGs demand for and access to SRHR.
3. To measure the effect of Balika Bodhu on empowerment of the MAGs;
4. To measure the effect of Balika Bodhu on social norm around SRHR of the MAGs;
5. To explore what works or does not work in promoting SRHR among the MAGs; how change happens or why change does not occur.

## Methods

**Fig 1:**
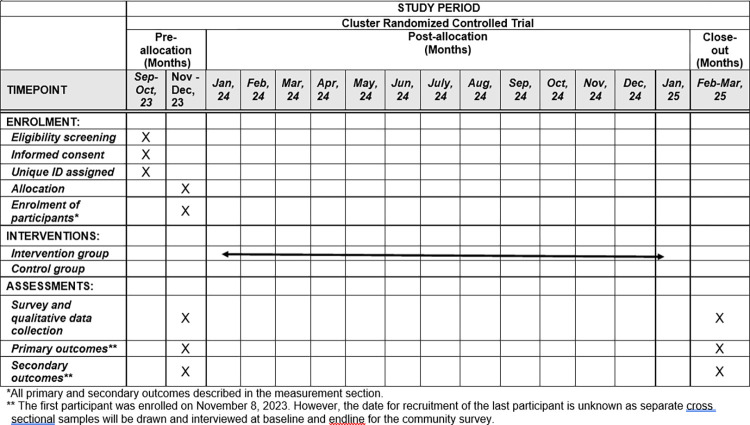
Schedule of enrollment, interventions, and assessments.

In developing this protocol, we followed the guidelines outlined in the Standard Protocol Items: Recommendations for Interventional Trials (SPIRIT) (S1 Checklist). The timeline for enrollment, interventions, and assessments are presented in Fig 1.

### Trial design

**Fig 2:**
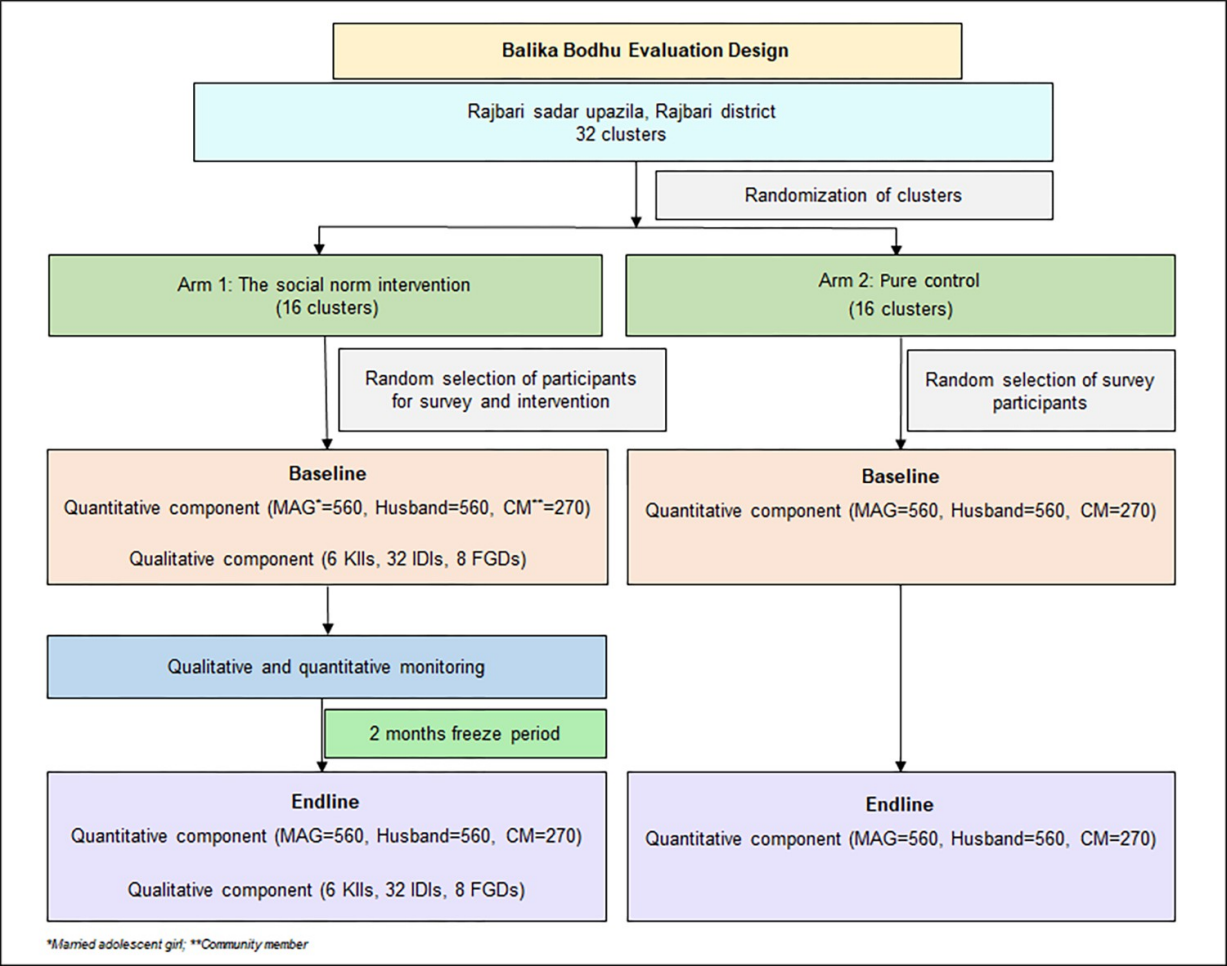
The Balika Bodhu evaluation design.

The Balika Bodhu evaluation trial employs a mixed-method, two-arm, Cluster Randomized Controlled Trial (CRCT) design (Fig 2). The arms are as follows:

**Arm 1**: The combined social norm and MAG empowerment intervention
**Arm 2**: Control

### Study site

Balika Bodhu will be implemented in the Rajbari Sadar upazila (sub-district) under the Rajbari district. We selected Rajbari district as the study site, because our focus is on married adolescent girls and Rajbari district has higher rate of child marriage (56%) than the national rate (59%) [3, 33]. According to Multiple Indicator cluster survey (MICS), Rajbari also reports higher than national rates of discrimination/harassment against women (26% vs. 11%) and justification of wife beating (42% vs. 25%). Additional advantage of Rajbari include a large number of villages necessary to accommodate the required number of clusters (32) with buffer zones.

### Cluster formation, randomization, allocation concealment and blinding

Villages within the Rajbari Sadar upazila will serve as clusters or the primary sampling units. The list of villages and their size will be obtained from the upazila statistics office. Any village/cluster with approximately 500 or more households will be considered eligible for inclusion in the study. For villages that share borders, only one eligible village will be considered as a potential cluster and all other villages surrounding the selected village will be treated as buffer zones, ensuring protection of clusters from contamination. This exercise will be undertaken for the whole study upazila. Three teams, each consisting of two members, will visit each of the eligible clusters, talk to local people, check the size of the village and decide which could be could be selected as potential study clusters and which could be potential buffers. We will then randomly select 32 clusters/villages from the final list of eligible clusters and assign them randomly to either the intervention or control group in a 1:1 ratio. The randomization process will be carried out by an independent statistician from icddr,b who will not be involved in the designing the study, recruitment of the study participants, implementation of the intervention or analysis of the data. This strategy will reduce any potential bias in randomization. No one will be blinded in this study.

### Study population

The study population will be married adolescent girls (MAGs) aged 15–19 years, their husbands, and community members (females and males) aged between 35 to 59 years.

### Inclusion criteria

The MAGs included in the study will be: i) aged 15–19 years, ii) currently married and residing with their husbands, and iii) both spouses have no plans to migrating out within the next 15 months. The husbands of the selected adolescent girls will also be included in the study. Polygamous marriages are rare in Bangladesh [34]. According to Kramer [35], fewer than 1% of Muslim men live with more than one spouse in Bangladesh. Therefore, this is not a major issue in the current study. In the unlikely event, where we encounter this, we will follow our stated sample selection procedure. Thus, one married adolescent girl will be selected first from a household. If there are more than one married adolescent girl in a household the participant will be selected randomly from among them. Once the married adolescent girl has been selected her husband will automatically become a potential participant of this study. Due to ethical and safety concerns, no more than one married adolescent girl will be selected from one household. Additionally, women and men aged 35–59 years in each cluster will be eligible to participate in the community survey. This group of participants will be included in this study to measure community’s perspective (attitudes and social norm) regarding SRHR of MAGs.

### Household enumeration, sampling and recruitment of study participants

Household enumeration will be conducted in each cluster to collect information such as gender, age, and marital status of the household members. Additionally, we will also ask whether any MAG is living with their spouses to determine their eligibility to participate in the study. In the large villages, enumeration will be stopped once we reach 500 households.

From the list of eligible MAGs, we will randomly select 35 MAGs from each cluster using a computer-generated sequence, overseen by the same independent statistician. The number 35 was determined, considering a 10% estimated non-response rate in the baseline survey and an additional 20% who may refuse to participate in the intervention, resulting in MAG and husband group sizes of 25 for the group sessions within each cluster. Baseline interviews will be conducted at the time of participant recruitment. If any selected MAG is found not to meet the eligibility criteria during screening at the baseline survey, she will be replaced by another randomly selected eligible MAG. In cases where a cluster contains fewer than the required number of eligible MAGs, a new neighborhood or *para* from the same village will be included as part of the cluster to achieve the required number of MAGs. If there is no remaining *para* for inclusion, a *para* from a bordering village will be included as part of the cluster.

The selected MAGs and their spouses will be enrolled in the study and included in the baseline survey. Following the baseline survey, the MAGs and their spouses from the intervention villages will be invited to participate in the Balika Bodhu intervention. All the MAGs and their husbands who were interviewed at baseline will also be interviewed at the endline.

For the community survey, separate cross-sectional samples will be drawn at baseline and endline. Lists of eligible community members aged 35–59 years will be obtained from the household enumeration. From each cluster, nine females and nine males making the total 576 will be randomly selected at baseline and at endline using a computer-generated sequence. Any community member not meeting the eligibility criteria (e.g., under or over aged, does not live in the cluster) during the screening at the time of the survey will be replaced by another randomly selected community member. No more than one MAG and/or community member from the same household will be included in any of the surveys.

The first participant was enrolled on November 8, 2023. However, the date for recruitment of the last participant is unknown yet as separate cross sectional samples will be drawn and interviewed at baseline and endline for the community survey.

### The social norm intervention

**Fig 3:**
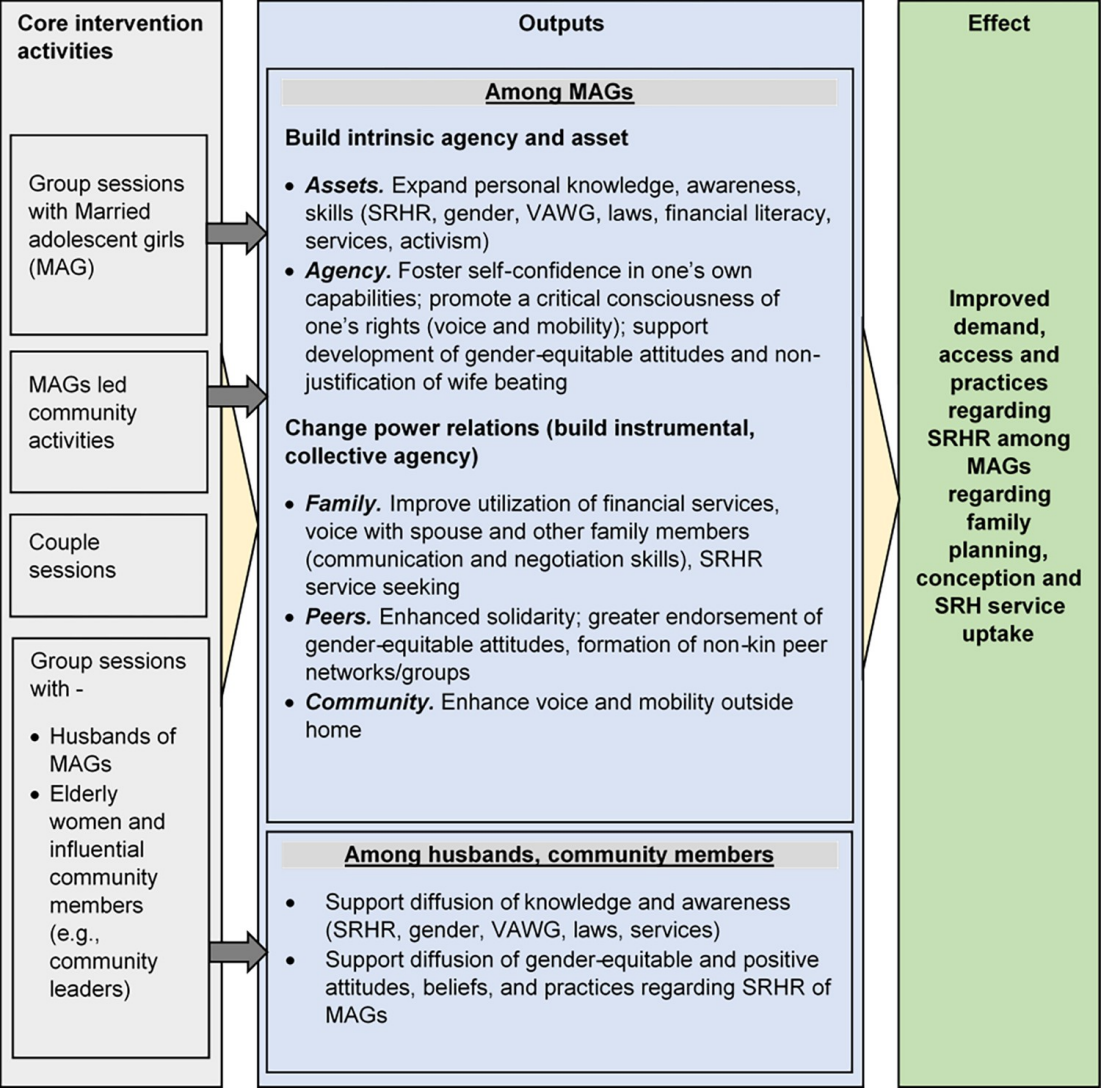
The Balika Bodhu Theory of Change (ToC).

Fig 3 depicts the Theory of Change (ToC) that the Balika Bodhu intervention is guided by. The ToC has been adapted from the Tipping Point Initiative’s Gender Empowerment and Women’s Voice Framework [36]. According to the framework, empowerment of MAGs refers to the expansion of their abilities to engage in, negotiate with, influence, control, and demand accountability from institutions that impact their lives. Accordingly, the intervention will focus on three domains of change, namely (1) individual agency that builds awareness and empowers MAGs with knowledge, skills and capabilities about equality, rights, SRHR, livelihood options, and social norms. Individual agency is manifested in women’s aspirations, resources, actions and achievements; (2) power relations through which MAGs navigate their lives (e.g., negotiating their SRH needs and rights with other social actors, including men; and taking greater charge of their body/sexuality); and (3) the structures that support promotion SRHR among MAGs.

The inputs (on the far left of Fig 3) illustrate the project components, which will impact each of these domains. At the individual level, through group sessions and activities, the intervention will build agency and assets of MAGs, and will change power relations in the family, and in the community. Relationally, the intervention will target the spouses of MAGs and community members to encourage more trusting relationships with the MAGs that support their SRHR and work in solidarity to achieve that goal.

In line with the ToC, the Balika Bodhu intervention will be developed by icddr,b heavily drawing on CARE’s IMAGINE model [27] that has been implemented in Bangladesh and Niger during 2016–2022 and on SAFE, a project spearheaded by icddr,b to promote SRHR and to address violence against women in the slums of Dhaka) [37]. Design of SAFE intervention was inspired by programmes such as Stepping Stones [38], Raising Voices [39] and a pilot intervention to promote gender equity among young men from low-income communities in Mumbai, India [40].

Balika Bodhu intervention will be implemented by Nari Maitree, an NGO working on women’s issues. The intervention will involve a 12-month gender transformative participatory programming. The intervention will target the MAGs, their spouses, elderly women and influential community members. Each group session will be facilitated by a trained gender-matched facilitator and a co-facilitator, while a community volunteer will serve as the gate-keeper.

Table 1 presents the details of the group sessions and other activities included in Balika Bodhu intervention. Thirty-six 2-hour weekly sessions will be conducted with the MAGs. The spouses of the MAGs will receive 20 two-hour bi-weekly sessions. The session topics will be broadly categorized into: i) Health and life skills covering topics such as sex and gender, human rights, marriage, SRHR, family and conjugal life and life skills; and ii) Business and entrepreneurship (only for the MAGs).

**Table 1:** Balika Bodhu intervention plan.

Theme/Topic	Session title		No. of sessions	Type of participants	
				MAG	Husband
**Session plan for MAGs and husbands**					
Introduction	Introduction to the project and training		1	√	√
***Theme*: *Health and life sessions***					
Sex and gender	Sex and Gender		1	√	√
Human rights	Human rights		1	√	√
Marriage and marital relationship	• Marriage, registration, dowry • Leaving wife, polygamy, and divorce		2	√	√
Sexual and reproductive health and rights (SRHR)	• Sexual and reproductive health and rights (SRHR) • Fertility and conception		2	√	√
Teenage first pregnancy	• Teenage first pregnancy—health perspective • Teenage first pregnancy—economic perspective • Teenage first pregnancy—impact cycle		3	√	√
Family planning (FP)	FP and contraception		2	√	√
Family and conjugal life	• Healthy relationships—sources of love, caring and support • Communication and negotiation • Ways to avoid mental confusion, contradiction, and turmoil • Anger management • Impact of gender discrimination and violation of human rights in marital and family life		6	√	√
Life skills	• Goal setting • Household budgeting		2	√	√
	• Activism and leadership skills		1	√	
	• Community action planning		1	√	
***Theme*: *Business and entrepreneurship sessions***					
Introduction to Planning	Introduction to Planning		1	√	
Planning for problems	Planning to resolve the problems		1	√	
Market Research and adding value	Market research and adding value		1	√	
Market information from the client	Collecting market information from the client		1	√	
Consider all options	Thinking alternative options		1	√	
Pros and cons of an alternative option	Pros and cons of an alternative option		1	√	
Taking smart risks	Taking smart risks		1	√	
Business plan	Business plan		1	√	
***Review sessions***	**-**		6	√	
**Session plan for couple, elderly women and influential community members**					
			Couples	Elderly women	Influential community members
Teenage first pregnancy	Teenage first pregnancy	1	√	√	√
Unplanned and unintended pregnancy and birth spacing	Unplanned and unintended pregnancy and pregnancy gaps	1	√	√	√
Action planning	Action planning	1	√	√	√
**Other activities**					
Community-level social norm activities	At least two community-level social norm activities, led by the MAG leaders, will be conducted in each cluster.				
Vocational training	MAG leaders and any other interested MAG group members will receive vocational training on income generation offered by the government				

A total of three 2-hour couple sessions will be held every two months, starting from the 6th month of the intervention. The session topics will include teenage pregnancy, unintended pregnancy and birth spacing, and action planning. Separate groups of elderly women and influential community members will receive three 2-hour bi-monthly sessions each. The topics and scheduling of these sessions will be the same as in the couple sessions.

In addition to group sessions, the MAGs demonstrating leadership qualities will be identified and trained to lead campaigns and activism. They will be put in contact with the other MAG groups and networks, mentored and provided with resources to execute at least two community-level social norm activities. These leaders will also receive training on income generation. Arrangements will be made so that the MAG leaders and any other interested MAG group members receive vocational training offered by the government.

Briefly, the key strategies for ensuring intervention fidelity will include the following: Recruitment of a monitoring officer; training of intervention implementers and the monitoring officer; ongoing monitoring of the intervention by the monitoring officer; weekly meetings of intervention implementing staff and the monitoring officer; implementation of an ongoing quantitative monitoring of the program; bi-weekly meetings between icddr,b and the implementing partner for analyzing quantitative monitoring data and other feedback from weekly field meetings; three rounds of qualitative monitoring involving group session observation, FGD with field staff and In-Depth Interviews with program participants; analysis of qualitative monitoring data and feedback to the implementing partner.

#### Monitoring of Balika Bodhu

Both qualitative and quantitative monitoring will be conducted to ensure the implementation fidelity of Balika Bodhu and documentation of any deviations. Number and duration of group sessions, attendance, and number of activities/events performed will be tracked by facilitator, cluster, and target group. These data involving bi-weekly reporting will be useful for any necessary course correction. A field-based female monitoring officer will be in charge of quantitative monitoring. She will receive a comprehensive training on the session manuals and quantitative monitoring of the intervention implementation. She will enter data using an offline-based software developed in SurveyCTO. A researcher will analyze these data on a regular basis. She will observe sessions on a regular basis and provide on-spot feedback to the facilitation team. She will also discuss the issues in weekly team meetings with the implementation team and weekly meetings between icddr,b and the implementing partner. The issues will be discussed and feedback will be provided to the implementation team and modification will be made to plan if needed.

The research team will conduct three rounds of qualitative monitoring. Table 2 presents the details of qualitative monitoring. Feedback from qualitative monitoring will help address any challenges and weaknesses in the intervention delivery and record any deviations from the charted program, which will be important to take into consideration during the interpretation of the results. Five sessions with the MAGs and three with the husbands will be observed in all three rounds. In addition, two sessions with the couples, and two with the community members will be observed in round 2 and 3. Five short interviews with the MAG group members and three with husbands will be conducted in all three rounds. One Focus Group Discussion (FGD) will be conducted with the facilitators and one with the co-facilitators in each round. Moreover, three community events will be observed at round 3.

**Table 2:** Qualitative monitoring of Balika Bodhu intervention.

Round of monitoring	Timing	Method	Target group	Number
Round 1	3^rd^ month into group sessions	Session observation	MAGs	5
			Husbands of MAGs	3
		Short interviews with the group members	MAGs	5
			Husbands of MAGs	3
		Focus Group Discussion (FGD)	Facilitators	1
			Co-facilitators	1
Round 2	6^th^ month into group sessions	Session observation	MAGs	5
			Husbands of MAGs	3
			Couples	2
			Community members	2
		Short interviews with the group members	MAGs	5
			Husbands of MAGs	3
		FGDs with the group session facilitators	Facilitators	1
			Co-facilitators	1
Round 3	9^th^ month into group sessions	Session observation	MAGs	5
			Husbands of MAGs	3
			Couples	2
			Community members	2
		Short interviews with the group members	MAGs	5
			Husbands of MAGs	3
		FGDs with the group session facilitators:	Facilitators	1
			Co-facilitators	1
		Observation of community event	Community members	3

#### Study outcomes and measurement

The primary outcome of this study is–instrumental agency of the MAGs in decision making regarding SRHR (i.e., FP use; timing of first conception; and number of children to have). Table 3 presents the summary of primary and secondary outcomes of this study and detailed description of the measurement of the outcomes have been presented in S1 File. Apart from attitudes, our study focuses on women’s experiences and their narratives. Thus, while attitudes and practices regarding SRHR will be measured among the MAGs, only attitudes regarding SRHR of MAGs will be measured among the husbands and community members. We will measure social norm using the community survey data. In addition, we will collect data on MAGs’ perspective on social norms, since this perspective may contribute to their practices.

**Table 3:** Different outcomes to be measured among different target groups of Balika Bodhu.

Sl	Outcome	Target population	Measurement	Questions/Scale used	Number of items/ questions	Reliability and validity test	Expected direction of change
**Primary outcome**							
P1	Instrumental agency of the MAGs in decision making regarding SRHR (i.e., FP use; timing of first conception; and number of children to have)	MAGs	Proportion of MAGs reported higher instrumental agency (using tertiles of score)	Questions will be framed around decision making regarding FP use, timing of first conception and number of children to have	6	-	Increase
**Secondary outcomes**							
S1	Agency of the MAGs related to women’s empowerment	MAGs	Proportion of MAGs reported higher intrinsic, instrumental and collective agency and critical consciousness (using tertiles of score)	Women’s Agency Scale 61 (WAS-61) [41] and Critical Consciousness Scale (CCS) [42]	61; 15	WAS-61: CFI = 0.94; TLI = 0.93; RMSEA = 0.04 CCS: CFI = .98; TLI = .97; RMSEA = .05	Increase
S2	Couple communication initiated by the MAGs regarding FP use, timing of first conception, and number of children to have	MAGs	Proportion of MAGs initiated communication with her husband regarding her choice of FP use, timing of first conception, and number of children to have.	Questions will be framed	3	-	Increase
S3	MAGs attempt to negotiate FP use, timing of first conception, and number of children to have as per her choice	MAGs	Proportion of MAGs attempted to negotiate her choice of FP use, timing of first conception, and number of children to have.	Questions will be framed	3	-	Increase
S4	Service uptake among the MAGs regarding abortion and post-abortion care, and help seeking after experiencing violence	MAGs	The proportion of MAGs who sought post-abortion care and the proportion of MAGs who sought help for physical and/or sexual intimate partner violence.	Questions will be framed	2	-	Increase
S5	Prevalence of intimate partner violence (IPV) among the MAGs	MAGs	Prevalence of intimate partner emotional, physical and sexual violence. Prevalence of economic coercion	Modified version of the Revised Conflict Tactic Scale [43]; Modified version of the ECS-20 [44]	15 20	- CFI = 0.95 TLI = 0.94 RMSEA = 0.03	Decrease
S6	Attitudes regarding agency of the MAGs regarding SRHR among the husbands	Husbands of MAGs	Proportion of husbands of the MAGs with positive attitudes regarding agency of the MAGs regarding SRHR	Questions will be framed	17	-	Increase
S7	Non-condoning attitudes of husbands of the MAGs regarding violence against women	Husbands of MAGs	Proportion of husbands of the MAGs with non-condoning attitudes regarding violence against women	Questions will be framed	6	-	Increase
S8	Attitudes regarding agency of the MAGs regarding SRHR among the community members	Community members	Proportion of husbands of the MAGs with positive attitudes regarding agency of the MAGs regarding SRHR	Questions will be framed	17	-	Increase
S9	Attitudes of community members regarding violence against women	Community members	Proportion of community members with positive attitudes regarding violence against women	Questions will be framed	6	-	Increase
S10	Social norms regarding SRHR of the MAGs	MAGs; Community members	Proportion of MAGs and community members with positive social norm regarding SRHR of the MAGs	Questions will be framed	28	-	Increase

### Quantitative component

Quantitative surveys will be conducted at baseline and endline of the cohort of MAGs and their spouses and from separate cross-sectional samples of adult males and females within the community in all the 32 study clusters.

As we aim to test the impact of different parts of the intervention on various populations, such as the empowerment component for married adolescent girls (MAGs), knowledge and attitudes for husbands of MAGs, and social norms for community members, we need distinct samples from each corresponding population. The sample size of the MAGs was determined based on the primary outcome, i.e., the ability to make choices regarding FP use, conception, and the number of children to have. We assumed an unknown prevalence rate of 50% for the instrumental agency of the MAGs in decision making regarding SRHR (as no information was available), an effect size of 15%, a group size of 22, an intra-cluster correlation of 0.05, a significance level of 5%, and a power of 80%. The 15% effect size was fixed based on the existing literature. A systematic review and meta-analysis by Lassi et al., [45] showed that sexual and reproductive health empowerment intervention increased ever use of contraception by 22%. In a study in Zimbabwe, female youth who participated in an intervention reported significant increases in condom negotiation self-efficacy compared with those in the control condition by 17% (AOR:1.17; 95% CI: 0.95–1.43) [46]. In a school-based program to increase girls’ empowerment and boys’ education about gender equity among youth (ages 10–16) in Kenya increased general self-efficacy to cope with stress and manage difficulties by 19 percentage points (mean score increase  =  .19, 95% CI: 0.08–0.39) [47]. Based on variation in effect size in different studies we considered 15% which is close to the lowest one. Considering these varying effect sizes in different studies and taking into account huge challenges commonly encountered in changing social norms and in empowering girls in a patriarchal setting we considered a more or less conservative effect size of 15% in our study. In the social context of Bangladesh with overtly restricted mobility on young married women, participation of married adolescents is likely to be very low in the intervention. Thus, we further considered a 10% non-response rate at baseline, 20% refusal rate among the MAGs interviewed at baseline to participate in the intervention, and another 15% dropout rate at endline for the MAGs, who participated in the intervention. This resulted in a required group size of 35 and a total sample size of 1,120 MAGs. This sample size allows us to measure changes in all other secondary outcomes of the study. The husbands of the selected MAGs will also be included in the study to measure the secondary outcomes related to their knowledge and attitudes regarding SRHR.

To assess changes in social norms surrounding MAGs’ consent and choice regarding SRH issues, we will survey the adult community members aged 35–59 years. Since the prevalence of positive social norms in this population is unknown, we assumed it to be 50%. Considering an effect size of 15%, a significance level of 5%, a power of 80%, and a non-response rate of 5%, we required a total sample size of 540 community members (270 females and 270 males) from 32 clusters. By dividing the numbers by the number of clusters and rounding the numbers up, the total sample size became 576 (nine females and nine males from each cluster).

#### Data collection

Three separate questionnaires will be developed in English. Most of the modules included in these questionnaires were previously pre-tested, piloted, validated and used by the research group in other studies in the country. We will use translated version of these modules in our questionnaires. The rest of the questionnaires will be developed in English and then translated into Bengali. Translation will be performed by one of the researchers and then checked by another researcher. We will make sure that at least one of them will be a mix-method researcher having long experience of developing appropriate survey tools for rural Bangladesh. All the three questionnaires will undergo pre-testing. All the three questionnaires will undergo pre-testing. The new modules not involving standard tools will undergo cognitive testing to assess: i) what does the respondent believe the question to be asking; ii) what do specific words and phrases in the question mean to the respondent; iii) whether question gathers intended information; etc. The responses will be reviewed and questions will be rephrased based on feedback received. Finalization of the questionnaires will occur following piloting of the questionnaires. The English version of the questionnaires will then be updated according to the Bengali version.

The data collection team at baseline and endline will consist of six female and six male members, two supervisors, and one survey coordinator, all with graduate degrees at a minimum. The team will receive an extensive 12-day participatory training covering a wide range of topics (e.g., gender, SRHR, empowerment, survey methodologies, including research ethics, questionnaires, use of tablets for data collection, ensuring data quality).

Data will be collected through face-to-face interviews conducted in Bengali by gender-matched interviewers. Three separate survey forms will be developed in SurveyCTO for the purpose. Prior to interview, oral informed consent will be obtained from the study participants. All interviews will be conducted privately, at a location convenient for the participants, using tablets. If a participant cannot be reached by the survey team after three visits during the entire data collection period, they will be considered unavailable.

#### Data quality monitoring and management

The supervisor will closely observe the interviews, take detailed notes, and address any issues during daily review sessions. Interviewers will randomly cross-check each other’s interviews and offer feedback. The survey coordinator will select interviewers for spot checks on a random basis. At the end of each business day, the collected data will be uploaded to a dedicated server, enabling researchers to promptly review it. Furthermore, a software-based data checking routine will be developed to swiftly identify inconsistencies in the data. A researcher will analyze the data on a weekly basis, identifying any inconsistencies and providing feedback to the survey team. Corrections will be made following standard procedures. To ensure quality, supervisors and the coordinator will revisit five percent of the study participants to administer a brief questionnaire. Additionally, researchers will make several field visits to observe interviews and provide feedback.

#### Cohort tracking

Cohort tracking is essential in a CRCT. In the intervention clusters, Nari Maitree will be in regular touch with the study participants. If someone is absent in any session, the session facilitators will contact them over phone and, if needed, through home visits. They will maintain the most updated contact details of the study participants. A research assistant will track the study participants in the control clusters calling them every three months to update their contact information. Reasons for any dropout will be recorded. Attempts will be made to conduct endline interviews with the participants even if they reside within the upazila after leaving the cluster they were recruited from. Study participants’ contact information will be stored separately from the survey data and kept under lock and key.

#### Data analysis

Items in the survey questionnaires will be recoded so that all are anchored at 0. For example, for partner communication, each item scored 1–4 in the questionnaire will be recoded to 0–3. The scales will be validated for the target population using exploratory factor analysis. The rotated factor loading of an item against all the suggested factors will be checked for non-loading (<0.35) and cross loading (loaded under two or more factors). Any factor with less than 3 items will be dropped. Internal consistency (or reliability) of a scale will be measured using Cronbach’s alpha (≥0.60 will be considered acceptable) and validity using the Kaiser-Meyer-Olkin (KMO) test (≥0.60 will be considered acceptable). Once the final scale is constructed, a summative score will be obtained for each scale, and the score will be divided into tertiles. For any scale, to keep the directions of all the items same, statements will be reversely coded as needed.

To ensure balance between the control and treatment groups, we will compare the basic background characteristics (including age, education, religion, and ethnic group) of the MAGs, their husbands, and community members using the baseline survey data. The baseline survey data will also be used to investigate the current status of empowerment and SRHR among the MAGs and correlates.

Descriptive analyses will be conducted to report the frequencies and percentages of different outcomes of interest. Statistical tests such as Chi-square, ANOVA, and t-tests will be performed to verify whether the study arms are well-balanced in terms of covariates. Linear and logistic regression analyses will be employed to identify the underlying factors contributing to the current situation in SRHR among MAGs.

An intention-to-treat (ITT) analysis will be used to assess the impact of Balika Bodhu using the baseline and endline data. All the MAGs enrolled at baseline and successfully interviewed at endline will be included in the analysis, regardless of their actual level of participation in the intervention activities. The impact of the intervention on primary and secondary outcomes will be assessed using regression coefficients obtained from regression analyses, binary or linear, as appropriate based on the type of outcome variable. This will measure the changes in outcomes in the intervention arm compared to the control arm. The statistical significance level will be set at p < .05 for all analyses. The data will be analyzed using STATA 16.

Data collection using Tablets prevent occurrence of unnecessary missing values. However, missing values may occur if any respondent decline to respond to a specific question or a section as a whole. We will impute missing values, where possible, using multiple imputation method.

### The qualitative component

The qualitative study will cover two purposively selected intervention clusters. Data will be collected through Key Informant Interviews (KIIs), In-depth Interviews (IDIs), and Focus Group Discussions (FGDs) (Table 4). While KIIs and FGDs will provide data on social norms and general practices around SRHR of the MAGs, IDIs will provide insights into individual perceptions, practices and experiences. All sources of data will help understand the impact of Balika Bodhu or the lack of it and the underlying factors.

**Table 4:** Qualitative methods and participants.

Method	Participants	Baseline	Endline
Key Informant Interviews	• Mothers-in-law of MAGs • NGO staff • Government community health workers (e.g., Family Welfare Visitor) • Village doctors/drug sellers/ traditional healers	6	6
In-depth interviews	MAGs	16	No. of MAGs and husbands retained in the cohort
	Husbands of MAGs	16	
Focus Group Discussion	MAGs	2	2
	Husbands of MAGs	2	2
	Elderly women aged 35–59 years	2	2
	Influential community members aged 35–59 years	2	2
**Total**		**46**	**46 (max)**

We will conduct a total of six KIIs with mothers-in-law (MiLs), Non-government Organization (NGO) staff implementing SRHR programs in the study area, Community Health Workers (CHWs), village doctors / traditional healers /drug sellers.

Sixteen IDIs will be conducted with the MAGs and 16 with their husbands from the cohort. Attempt will be made to interview the same MAGs and husbands at baseline and endline to better understand change. FGDs will be conducted separately with MAGs, their husbands and elderly women and influential community members in each village making the total eight.

Each KII, IDI, and FGD will take around one and a half hours. All interviews will be digitally recorded with consent from the participants. We will collect qualitative data at baseline and endline. Table 4 describes the methods, participants, procedure, and tentative sample size of the qualitative study.

#### Data collection and processing

Qualitative data collectors with Masters degree in social science will receive an 8-day training on gender, rights, empowerment, SRHR, communication, negotiation, decision-making, social norms, qualitative research methods, and research ethics. Interviewers will be gender-matched with the study participants. IDIs will be conducted with the same MAGs at baseline and endline. All interviews and FGDs will be digitally recorded upon receipt of informed consent from the participants. The recorded data will be transcribed verbatim.

#### Data analysis

We will combine the intense, within-case Narrative Analysis with the across-case approach of Grounded Theory in our qualitative data analysis. Narrative analysis will be used to examine each IDI for a core narrative or ‘story’ about attitudes regarding and experiences of the MAGs and their husbands in the domains of our interest. We will then compare these core narratives across participants to identify both unique and common elements within the narratives. Narrative analysis will reveal, for instance, the baseline status of the MAGs and their husbands in the stated areas. A comparison of the IDIs of the same MAGs and their husbands at baseline and endline will offer valuable insights into change that relates to Balika Bodhu.

### Sources of bias and mitigation strategies

This study involves randomizing groups or clusters of subjects rather than individual subjects, which can introduce unique sources of bias. Also, this is an unblinded trial which could introduce bias in outcome assessment. Though the nature of the study does not permit us to go for a blinded trial, we have secured a few strategies to mitigate these biases:

- Lack of Blinding: Unblinded assessments, where evaluators or participants are aware of treatment allocations, can lead to various types of bias. To mitigate this, we will pre-register the trial’s design, methods, and outcomes to increase transparency. Also, to reduce bias, two different organizations, Nari Maitree and icddr,b are implementing the intervention and evaluating it respectively.
- Selection bias: This can arise if the randomization process is not properly controlled. To mitigate this, an independent statistician, who will not be involved in other aspects of the study, will oversee the randomization to ensure it is conducted properly and without bias.
- Contamination: This occurs when interventions in treatment clusters affect individuals in control clusters. To prevent this, we will physically separate clusters to minimize interaction between them by ensuring buffer zones between selected clusters.
- Confounding: This type of bias occurs when unmeasured variables influence the study’s outcome. To control for this, we will collect information on a broad range of potential confounding variables, allowing us to adjust for them during analysis.
- Attrition bias: This happens when there is an unequal loss of participants across clusters, potentially impacting the outcomes. To address this, we will use intention-to-treat analysis to ensure that all participants are included in the final analysis based on their original assignment.

### Ethics and dissemination

This research follows the CIOMS International Guidelines for Ethical Review of Epidemiological Studies for both quantitative and qualitative aspects and adheres to the WHO ethical recommendations for investigating violence against women. Ethical approval for the study was obtained from the Institutional Review Board (IRB) at icddr,b (PR-22145; version No. 4.0; version date October 23, 2023). Participation in the study is entirely voluntary. Prior to the interview, individual oral consent will be obtained by the interviewer from each study participant. Written consent will not be used in this study mainly for two reasons. First, because this study addresses sensitive topics such as sexual and reproductive coercion and violence, involving vulnerable populations. In keeping with the guidelines for researching sensitive issues we ensure the participants confidentiality and anonymity. Asking for written consent is in conflict with the strategy of ensuring anonymity and confidentiality. Women may not feel safe in signing a document revealing her identity in a sensitive study. In contrast, oral consent can create a safer and more comfortable environment for the participants. By choosing oral consent, we aim to protect the participants for potential leakage of identity. Secondly, written consent is also being avoided due to low literacy levels among many of the study participants. Participants will receive comprehensive oral information regarding the study’s purpose, nature, process of interview, anticipated risks and benefits, sensitivity, confidentiality measures, future use of information, and the voluntary nature of their participation. Once the participant gives consent to take part in the study, the interviewer will record the response and sign off the consent form. The supervisor will be involved in the process and will sign off the consent form as a witness. The MAGs below 18 will be recognized as "mature minors" in this study. Informed consent will be sought from them in accordance with the guidelines for adolescent health research outlined by the National Commission for the Protection of Human Subjects of Biomedical and Behavioral Research and the Society for Adolescent Medicine [48, 49].

All participants in the study will be assigned a unique code, and any personally identifiable information will be stored in a separate file accessible only to the research team. This information will be utilized by the research team for participant tracking throughout the intervention period and for communication during the endline survey. The study findings will be shared through publications in peer-reviewed journals as well as through conferences and workshops involving stakeholders at the national and international levels who are involved in community-based healthcare delivery. Careful measures will be taken to present the research results in a sufficiently aggregated manner to safeguard the anonymity of all participants.

## Discussion

Balika Bodhu will assess whether and how social norms and empowerment of MAGs can support the promotion of SRHR among MAGs. The existing literature [18, 19] emphasizes the importance of addressing social norms to enhance the demand, access, and practice of positive behaviors regarding SRHR among MAGs. Empowerment of women and girls is widely recognized to enhance SRHR [50–52]. However, a rigorously evaluated intervention combining both is rare [53–55]. Even more rare are empowerment interventions that include all its critical elements [56]. Thus, the development, implementation, and rigorous evaluation of Balika Bodhu will address an important gap in the literature. The novelty of this study lies in: (1) combining social norms intervention with a comprehensive empowerment intervention that includes all its critical elements to address SRHR among married adolescent girls, a particularly vulnerable group commonly experiencing denial of SRHR; and (2) conducting a rigorous evaluation.

### Challenges and limitations

The total sample size was 2816, which is quite high but manageable. The budget has been carefully allocated to cover all data collection costs, including travel, communication (mobile phone cards), and per diem for the data collectors. The intervention is designed to benefit the participants by strengthening their knowledge and skills. In view of this, no per diem or compensation will be given for attending the sessions. However, if necessary, the participants will be provided with transport to attend the sessions. In addition, a token gift will be given to session participants. This strategy has been mentioned in the revised manuscript. The study participants will be interviewed at home or in a nearby place of her/his choice and no compensation will be given.

It is possible that participation in the intervention will not be optimal. The MAGs may not attend the sessions due to: (1) disapproval by themselves; (2) disapproval by their husbands and/or the mothers-in-law; (3) household chores; (4) distance to the session venue, as some of the clusters are formed combining 3–4 villages to ensure required number of MAGs in each cluster; (5) current pregnancy and/or having young child; etc. The husbands of MAGs may not attend the sessions due to: (1) disapproval by themselves; (2) disapproval by parents; (3) unavailability due to profession; etc. High rates of dropout and loss to follow-up can potentially impact the validity of the results. To mitigate these issues, the importance of compliance will be clearly explained to the participants. A rigorous follow-up procedure will also be established to encourage participation, monitor, and track the participants. This may involve regular check-ins and reminders to encourage their continued involvement; and union-level meetings with the chairman, members and local influential persons and village-level meetings with the participants, their family members, local influential persons to encourage their participation. Dose-response analysis will be employed to assess the full potential of the intervention when the study participants attend all or a higher number of sessions planned.

## Conclusion

Ensuring SRHR and equitable access to a comprehensive range of SRH services for females is of utmost importance for individual development and societal well-being. If Balika Bodhu proves effective, it will have significant implications for policies and programs in this field. Otherwise, it will send a valuable message to program developers and policymakers, highlighting aspects to be avoided when aiming to enhance the demand for and access to SRHR among MAGs. In either scenario, this study will significantly contribute to expanding the existing knowledge base by providing insights into effective approaches and potential pitfalls in improving demand, access, and practices related to SRH among MAGs.

## Supporting information

S1 Checklist SPIRIT checklist. (DOC)

S1 File Measurements of primary and secondary outcomes. (DOCX)

S1 Protocol Study protocol. (PDF)
