# Supplementary material for: A protocol for a cluster randomized controlled trial to assess the impact of Balika Bodhu: A combined empowerment and social norm based sexual and reproductive health and rights intervention for married adolescent girls in rural Bangladesh
Source: PLoS One. 2024 Aug 23;19(8):e0304988. doi: 10.1371/journal.pone.0304988 (PMC11343452; doi:10.1371/journal.pone.0304988)
Supplement: S1 File — (DOCX) [file pone.0304988.s002.docx]

**Measurements** **of primary and secondary outcomes**

Outcomes P1 and outcomes S1-S5 will be assessed using data from the MAG survey, while outcomes S6-S7 will be assessed using data from the husband survey and S8-S9 using data from the community survey. Outcome S10 will be assessed using data from both the MAG and community surveys.

# Primary outcome

**Outcome P1. Instrumental agency of the MAGs in decision making regarding SRHR (i.e., FP use; timing of first conception; and number of children to have)**

The MAGs will be asked about the main decision maker regarding: (a) FP use; (b) timing of first conception; and (c) number of children to have. The response options include ‘1 = the MAG herself’, ‘2 = husband’, ‘3 = jointly’, and ‘4 = someone else’. A follow up question, ‘what kind of joint decision it was’, will be asked if the response is ‘jointly’. The response options include ‘1 = he voiced his choice and I agreed’, ‘he voiced his choice and I had to agree’, and ‘3 = I voiced my choice and he agreed’. A MAG will be considered agentic in decision making regarding a specific theme of SRHR and will be coded as ‘Yes = 1’ if she decided herself or if it was a joint decision without any use of force on her, and ‘No = 2’ otherwise. We will generate a summative score, where a higher score will refer to higher agency.

# Secondary outcomes

**Outcomes S1. Agency of the MAGs related to women’s empowerment**

We will measure MAGs’ empowerment in terms of intrinsic, instrumental, and collective agency, and critical consciousness. The intrinsic, instrumental, and collective agency of the MAGs will be measured using the Women’s Agency Scale 61 (WAS-61) [1] and critical consciousness using by Critical Consciousness Scale (CCS) [2].

**Intrinsic agency:** We will assess women’s intrinsic agency using a validated, 29-item three-dimensional measure that includes women’s intrinsic voice and mobility, gender equitable attitudes, and non-justification of wife beating [1].

To assess intrinsic voice and mobility, nine items will assess women's comfort levels (1 = not at all to 3 = very comfortable) in activities such as going to places alone (e.g., the 'home of a relative') and expressing opinions with community members, including government officials, NGO representatives, and community leaders. A summative score will be calculated, with a higher score indicating higher intrinsic voice and mobility.

Gender equitable attitudes will be assessed through 14 items measuring women’s agreement (1 = strongly disagree to 4 = strongly agree) with statements such as 'a woman should obey her husband. Negatively valanced items will be reverse-coded, ensuring that a higher score reflects a stronger endorsement of more equitable attitudes.

The non-justification of wife beating will be assessed using six items that capture responses (1 = strongly disagree to 4 = strongly agree) to the question, ‘does a man have a good reason to hit his wife?’ in scenarios such as if she 'disobeys him' or 'rudely argues with him.' These items will also be reverse-coded, so a higher score indicates stronger non-justification of wife beating.

**Instrumental agency:** We will assess women’s instrumental agency through a validated, 17-item three-dimensional measure that measures women’s utilization of financial services, expression of voice with spouse, and voice and mobility outside the home.

Five items, such as ‘depositing money’ and ‘checking account’, will be used to measure women’s frequency of using financial services (1 = never to 4 = often). A summative score will be obtained, with a higher score indicating more frequent use of financial services.

Three items will capture women’s influence level (1 = none to 4 = total) with their spouse about ‘how to spend money your husband gives you’ and ‘how to spend your husband’s earnings’ and their frequency of expressing opinions (1 = never to 4 = often) with husbands. A summative score will be obtained, with a higher score indicating a higher level of influence on husband.

Nine items will be used to measure women’s frequency (1 = never to 4 = often) of activities such as going places like ‘the home of a relative’ and expressing opinions with community members, including government officials and community leaders. A summative score will be calculated, with a higher score indicating more frequent mobility and expressing opinions with community members.

**Collective agency:** We will assess women’s collective agency regarding influence in the community using an eight-item validated scale and regarding women’s leadership in groups using a validated seven-item scale [1]. The participants will report their agreement (1 = totally disagree to 4 = totally agree) with statements like ‘women like me can really understand what is going on with my community’ and ‘I am often a leader in groups’. Summative scores will be obtained, with higher scores indicating stronger collective agency.

**Critical consciousness**: Critical Consciousness (CC) is conceptualized to consist of two sub-components. One of them is critical reflection, which will encompass the ability to critically reflect on perceived societal inequalities as well as the authorization of societal equality. The second component is critical action, which encompasses individual and/or collective action taken to change perceived social inequalities [3]. We will measure critical consciousness of the MGAs using the validated 15-item Critical Consciousness Scale (CCS) [2]. Ten critical reflection related items will be used to measure perceived inequality and egalitarianism. A typical example includes ‘women have fewer opportunities than men’. The response options will be: strongly agree, agree, disagree, and strongly disagree. Another five items will be used to evaluate critical action, or the extent of their involvement in individual and/or collective efforts aimed at producing socio-political change, with response options – not at all, rarely, sometimes and often. A summative score will be calculated, with a higher score indicating a higher critical consciousness.

**Communication, negotiation, and Service uptake among the MAGs**

The MAGs will be asked about their communication, and negotiation regarding FP use, timing of first conception, number of children to have. They will also be asked regarding SRH service uptake. The questions will heavily draw on the standard Demographic and Health Survey questionnaires [4], SAFE [5], and IMAGINE questionnaires.

**Outcome S2. Couple communication initiated by the MAGs regarding FP use, timing of first conception, and number of children to have**

The MAGs will be asked about any communication with husband regarding FP use, first conception, and the number of children they want to have. The response options will include ‘1 = Yes’ and ‘2 = No’. They will also be asked about who initiated the conversation, with the response options ‘1 = herself’, ‘2 = husband’ and ‘3 = don’t remember’. Separate variables will be created from these responses by recoding ‘herself’ as ‘1’; ‘husband’ as ‘0’ and ‘don’t remember’ as ‘missing value’. A summative score will be obtained by adding the number of conversations she initiated on different SRHR issues, with a higher score indicating higher initiated communication.

**Outcome S3. The MAGs’ attempt to negotiate choice of FP use, timing of first conception, and number of children to have**

The MAGs will be asked what happened if there was any mismatch between her and her husband’s choices regarding the above mentioned SRHR themes. We will assess if a MAG attempted to negotiate in such a case and if her husband tried to convince her to accept his own choice. In both cases, an attempt to negotiate will be coded as ‘1’, and ‘0’ otherwise. A summative score will be calculated by accumulating the number of SRHR themes where she attempted to negotiate with a higher score indicating higher attempts.

**Outcome S4. Abortion and post-abortion service uptake and help seeking by physically and/or sexually abused MAGs**

If any MAG reports having an induced abortion/MR, she will be asked about the venue, the service received, provider, and the method of abortion. The MAGs with an abortion will be asked about post-abortion care. The MAGs who report experiencing any physical and/or sexual intimate partner violence will be asked about their help seeking behavior. The results will be presented in frequencies and percentages.

**Outcome S5. Intimate partner violence (IPV)**

The MAGs’ experiences of IPV (i.e. controlling behavior and physical and sexual violence IPV) will be assessed using a modified version of the Revised Conflict Tactic Scale [6]. The scale is the most widely used measure of IPV containing direct and behaviorally explicit questions in order to reduce variation in the interpretation and understanding of what violence comprises of. The questions will capture behaviors that indicate both moderate and severe physical violence (e.g., slapping, pushing, hitting with a fist, kicking, dragging, choking, burning, or threatening with a weapon), as well as sexual violence (e.g., forced sexual intercourse or other sexual acts).

To measure economic coercion, we will use a modified version of the ECS-20 [7]. Typical items include—your husband or any other family member ever and in last 12 months: has disallowed you to go to work, school or training, or to engage in any home-based income generating activity. Other items include being told that you could work outside the home only if you kept up with household chores or that you could earn income only by working from home, among others. A woman will be considered exposed to a specific type of IPV if she responds ‘yes’ to any of the items related to that specific type of IPV and will be coded ‘1 = Yes’, and ‘0 = No’ otherwise.

**Attitudes of husbands of the MAGs and the community members**

The husbands of the MAGs and community members will be asked about their attitudes regarding agency of the MAGs regarding SRHR, violence against women. The questions will heavily draw on the standard Demographic and Health Survey questionnaires [4], SAFE [5], and IMAGINE questionnaires.

**Outcomes S6 and S8. Positive attitudes of the husbands and the community members regarding agency of the MAGs around SRHR**

The attitudes of husbands of the MAGs and community members about agency of the MAGs around SRHR will be measured separately using 12 items. A typical example includes ‘it should be a wife’s decision when to have a child’. The participants will report their agreement on a 4-point Likert scale (1 = strongly agree to 4 = strongly disagree). A summative score will be obtained, divided into tertiles. Negatively valanced items will be reverse-coded, so a higher score indicates more positive attitudes.

**Outcomes S7 and S9. Positive attitudes of the husbands and the community members regarding violence against women**

Attitudes of the husbands of the MAGs and the community members regarding violence against women will be measured separately using six items. A typical example includes ‘there are times when a woman deserves to be beaten’. The participants will report their agreement on a 4-point Likert scale (1 = strongly agree to 4 = strongly disagree). A summative score will be calculated, with a higher score indicating more positive attitudes.

**Outcome S10. Community social norms related to consent and choice of the MAGs regarding their SRH**

We will measure social norms around consent and choice of the MAGs in SRH following Cialdini [8] and Bicchieri [9, 10]. The statements to capture social norms around consent and choice of the MAGs regarding their SRH will be framed considering Cialdini’s [8] and Bicchieri’s [9, 10] theories of social norms and validated for the target population. The theory of social norms is a theory of what motivates collective patterns of behavior [11]. It tries to answer a very basic question – why do people do what they do? The key concepts in this theory include empirical and normative expectations.

Empirical expectations refer to ‘what most people in her/his village will do in a specific circumstance’. A typical example includes: ‘do you strongly agree/agree/disagree/strongly disagree with this statement - most of the married adolescent girls in your village achieve their aspiration regarding their desired method of contraception’.

Normative expectations refer to ‘what most people in her/his village expect others to do in a specific circumstance’. A typical example includes: ‘do you strongly agree/agree/disagree/strongly disagree with this statement - most of the husbands of married adolescent girls in your village will approve if their wives try to achieve their aspiration regarding their desired method of contraception’.

Social norms around SRHR of the MAGs in the domains such as FP, conception, number of children to have, abortion, and SRH service uptake will be assessed separately using data from the MAG and community surveys. A set of statements will be used to assess the participants’ level of agreement collected on a Likert scale (1 = totally disagree to 4 = totally agree). A scale will be constructed and validated using factor analysis. A summative score will be calculated, with a higher value referring to stronger positive social norm.

# References

1. Yount KM, Khan Z, Miedema S, Cheong YF, Naved RT. The Women's Agency Scale 61 (Was-61): A comprehensive measure of women's intrinsic, instrumental, and collective agency. Instrumental, and Collective Agency (August 9, 2020). 2020.

2. Welch JC, Ezeofor I, Shin RQ, Smith LC. Development and Validation of the Critical Consciousness Scale. 2014.

3. Freire P. Pedagogy of the oppressed. New York, NY: Continuum.1993.

4. National Institute of Population Research and Training (NIPORT) and ICF. Bangladesh Demographic and Health Survey 2017-18. Dhaka, Bangladesh, and Rockville, Maryland, USA: 2019.

5. Naved RT, Mamun MA, Mourin SA, Parvin K. A cluster randomized controlled trial to assess the impact of SAFE on spousal violence against women and girls in slums of Dhaka, Bangladesh. PLoS One. 2018;13(6):e0198926.

6. Straus MA, Hamby SL, Boney-McCoy S, Sugarman DB. The revised conflict tactics scales (CTS2) development and preliminary psychometric data. Journal of family issues. 1996;17(3):283-316.

7. Miedema SS, Cheong YF, Naved RT, Yount KM. Development and validation of the Economic Coercion Scale-20 (ECS-20): A short-form of the ECS-36. PLoS one. 2023;18(10):e0287963.

8. Cialdini RB, Trost MR. Social influence: Social norms, conformity and compliance. 1998.

9. Bicchieri C. The grammar of society: The nature and dynamics of social norms: Cambridge University Press; 2005.

10. Bicchieri C. Norms, conventions, and the power of expectations. Philosophy of social science: A new introduction. 2014;208.

11. Bicchieri C. Measuring Social Norms University of Pennsylvania, Penn Social Norm Group. Available from: <http://docplayer.net/38473609-Measuring-social-norms-cristina-bicchieri-university-of-pennsylvania.html>?
