# Supplementary material for: A protocol for a cluster randomized controlled trial to assess the impact of Balika Bodhu: A combined empowerment and social norm based sexual and reproductive health and rights intervention for married adolescent girls in rural Bangladesh
Source: PLoS One. 2024 Aug 23;19(8):e0304988. doi: 10.1371/journal.pone.0304988 (PMC11343452; doi:10.1371/journal.pone.0304988)
Supplement: S1 Protocol — (PDF) [file pone.0304988.s003.pdf]

icddr,b	**RRC APPLICATION FORM**

RESEARCH PROTOCOL
Number: PR-22145
Version No. 4.00
Version date: 23-10-2023

FOR OFFICE USE ONLY

**RRC Approval:**	☒ Yes	☐ No	Date:17-12-2022
ERC Approval:	☒ Yes	☐ No	Date:22-01-2023
AEEC Approval:	☐ Yes	☐ No	Date:
External IRB Approval	☐ Yes	☐ No	Date:

Name of External IRB: ____________________

Will the protocol be submitted for expedited review? ☐ Yes ☒ No

If yes, please check all that apply:

- ☐ Outbreak investigation
- ☐ Secondary Analysis
- ☐ Formative research
- ☐ Short surveys
- ☐ Pilot Study
- ☐ Student protocol
- ☐ Observational study
- ☐ Approved by external IRB (local or abroad) except Randomized Clinical Trial
- ☐ Others (explain the justifications):

***** Cover Letter to RRC/ERC Chairperson through SDD must be attached.***

Protocol Title:* (maximum 250 characters including space) Development and testing of Balika Bodhu: a social norm intervention to address denial of sexual and reproductive health and rights to married adolescent girls in rural Bangladesh

Short Title: (maximum **100** characters including space) Development and testing of Balika Bodhu

Key Words:* Married adolescent girls, sexual and reproductive health and rights, family planning, conception, SRH service uptake, domestic violence, consent and choice, empowerment, agency, social norms

Name of the Research Division Hosting the Protocol:*

- ☐ Health Systems and Population Studies Division (HSPSD)
- ☐ Nutrition and Clinical Services Division (NCSD)
- ☐ Infectious Diseases Division (IDD)
- ☒ Maternal and Child Health Division (MCHD)
- ☐ Laboratory Sciences and Services Division (LSSD)
- ☐ Other (specify) ____________________

Has the Protocol been Derived from an Activity: * ☒ No ☐ Yes (please provide following information):

Activity No. :
Activity Title:
PI:
Grant No.: Budget Code: Start Date: End Date:

**icddr,b Strategic Priority/ Initiative (SP 2019-22):* (**check all that apply**)**	
☐ Reducing maternal, neonatal and child mortality and improving the well-being of women, children and adolescents<br>☐ Preventing and treating maternal and childhood malnutrition<br>☐ Detecting and controlling enteric and respiratory infections<br>☐ Detecting and controlling emerging and re-emerging infections<br>☒ Achieving universal health coverage	☒ Achieving gender equality and promoting sexual and reproductive health and rights<br>☐ Examining the health consequences of and adaptation to climate change<br>☐ Preventing and treating non-communicable diseases<br>☐ Others (specify)<br>______________________________
**Research Phase (4 Ds):* (**check all that apply**)**<br>☒ Discovery<br>☒ Development	☒ Delivery<br>☒ Evaluation of Delivery
**Anticipated Impact of Research:* (**check all that apply and please provide details below**)**<br>☒ Knowledge Production<br>☒ Capacity Building	☒ Informing Policy<br>☒ Health and Health Sector Benefits<br>☒ Economic Benefits

Please provide details here:

The study will generate knowledge on barriers experienced by married adolescent girls (MAGs) in demanding, accessing and practicing their rights regarding their sexual and reproductive health (SRH), including family planning (FP) use, conception, and seeking health care relating to SRH. We will also explore the underlying factors influencing such barriers. The study will develop and test a social norms intervention that addresses the gaps in programming and in the literature combining a comprehensive empowerment programme for MAGs with engagement of other stakeholders to increase demand, access and practices of positive behaviours in relation to sexual and reproductive health and rights (SRHR) among these girls. We will collaborate with a local organization, who usually work on empowerment and SRHR of women, for implementing the intervention and will build their capacity as well. All the information generated will inform the program and policy about the barriers and potential solutions to improve demand, access and practices of SRHR among the MAGs.

**Which of the Sustainable Development Goal This Protocol Relates to?:* (**check all that apply**)**	
☐ 1. End poverty in all its forms everywhere<br>☐ 2. End hunger, achieve food security and improved nutrition and promote sustainable agriculture<br>☒ 3. Ensure healthy lives and promote well-being for all at all ages<br>☐ 4. Ensure inclusive and equitable quality education and promote lifelong learning opportunities for all<br>☒ 5. Achieve gender equality and empower all women and girls<br>☐ 6. Ensure availability and sustainable management of water and sanitation for all<br>☐ 7. Ensure access to affordable, reliable, sustainable and modern energy for all<br>☐ 8. Promote sustained, inclusive and sustainable economic growth, full and productive employment and decent work for all<br>☐ 9. Build resilient infrastructure, promote inclusive and sustainable industrialization and foster innovation<br>☐ 10. Reduce inequality within and among countries<br>☐ 11. Make cities and human settlements inclusive, safe, resilient and sustainable<br>☐ 12. Ensure sustainable consumption and production patterns<br>☐ 13. Take urgent action to combat climate change and its impacts<br>☐ 14. Conserve and sustainably use the oceans, seas and marine resources for sustainable development<br>☐ 15. Protect, restore and promote sustainable use of terrestrial ecosystems, sustainably manage forests, combat desertification, and halt and reverse land degradation and halt biodiversity loss<br>☐ 16. Promote peaceful and inclusive societies for sustainable development, provide access to justice for all and build effective, accountable and inclusive institutions at all levels<br>☐ 17. Strengthen the means of implementation and revitalize the global partnership for sustainable development	
**Does this Protocol Use the Gender Framework?*** (Please visit: http://shetu.icddrb.org/index.php?option=com_content&view=article&id=265&Itemid=677 for Gender Alanysis Tool with instructions)	☒ Yes (please complete Gender Analysis Tool)<br>☐ No
If 'no' is the response, its reason(s) in brief:	
**Will this Research Specifically Benefit the Disadvantaged (**economically, socially and/or otherwise**):**	☒ Yes<br>☐ No
**Does this Protocol use Behaviour Change Communication?**	☒ Yes<br>☐ No

**Principal Investigator (Should be icddr,b staff):*** Sex ☒ Female ☐ Male<br><br>**Ruchira Tabassum Naved, PhD**<br><br>**Address:** (provide full official address including land phone no(s), Extension no.(if any), cell phone number and email address):<br>Emeritus Scientist<br>HSPSD Administration<br>Health Systems and Popualtion Studies Division<br>Extension: 2234<br>Mobile: 01713040946<br>Email: ruchira@icddrb.org<br><br>Do you have ethics certification? ☐ No ☒ Yes (please attach in your CV below)<br>Do you have RBM training certification? ☐ No ☒ Yes (please attach the certificate with CV below)	Primary Scientific Division of the PI<br><br>MCHD
**Co-Principal Investigator(s) Internal**: Sex ☐ Female ☒ Male<br><br>**Md. Mahfuz Al Mamun**<br><br>**Address:** (provide full official address including land phone no(s), Extension no.(if any), cell phone number and email address):<br>Assistant Scientist<br>Gender, Equity and Rights Research Group<br>Health Systems and Popualtion Studies Division<br>Extension: 2202<br>Mobile: 01713040947<br>Email: mahfuzmamun@icddrb.org<br><br>Signature or written consent of Co-PI:<br>__________________<br>(electronic signature or email or any sort of written consent)<br>[if more than one, please copy and paste this row for additional Co-PIs]<br><br>Do you have ethics certification? ☐ No ☒ Yes (please attach in your CV below)<br>Do you have RBM training certification? ☐ No ☒ Yes (please attach the certificate with CV below)	Primary Scientific Division/ Programme of the Co-PI<br>MCHD<br><br>__________________<br>Approval of the Respective Senior Director/ Programme Head<br><br>(Signature)

**Co-Investigator(s) - Internal:** Sex ☒ Female ☐ Male<br><br>**Aloka Talukder**<br><br>**Address** (Position, phone no, extension no, cell, and email address**):**<br>Research Investigator<br>Gender, Equity and Rights Research Group<br>Health Systems and Population Studies Division<br>Extension: 2203<br>Mobile: 01795975212<br>Email: aloka.talukder@icddrb.org<br><br>Signature or written consent of Co-I: ___________________<br>(electronic signature or email or any sort of written consent)<br>[if more than one, please copy and paste this row for additional Co-Is<br><br>Do you have ethics certification? ☐ No ☐ Yes (please attach in your CV below)<br>Do you have RBM training certification? ☐ No ☐ Yes (please attach the certificate with CV below)	Primary Scientific Division of the Co-I<br><br>MCHD<br><br>Approval of the Respective Senior Director/ Programme Head<br>(Signature)
**Co-Investigator(s) - Internal:** Sex ☐ Female ☒ Male<br><br>**Sultan Mahmud**<br><br>**Address** (Position, phone no, extension no, cell, and email address**):**<br>Research Investigator<br>Gender, Equity and Rights Research Group<br>Health Systems and Population Studies Division<br>Extension: 2202<br>Mobile: 01738761160<br>Email: sultan.mahmud@icddrb.org<br><br>Signature or written consent of Co-I: ___________________<br>(electronic signature or email or any sort of written consent)<br>[if more than one, please copy and paste this row for additional Co-Is<br><br>Do you have ethics certification? ☐ No ☐ Yes (please attach in your CV below)<br>Do you have RBM training certification? ☐ No ☐ Yes (please attach the certificate with CV below)	Primary Scientific Division of the Co-I<br><br>MCHD<br><br>___________________________<br>Approval of the Respective Senior Director/ Programme Head<br><br>(Signature)

**Co-Investigator(s) - Internal:** Sex ☐ Female ☒ Male<br><br>**Raafat Hassan**<br><br>**Address** (Position, phone no, extension no, cell, and email address**):**<br>Research Investigator<br>Gender, Equity and Rights Research Group<br>Health Systems and Population Studies Division<br>Extension: 2202<br>Mobile: 01912907953<br>Email: Raafat.Hassan@icddrb.org<br><br>Signature or written consent of Co-I: ___________________<br>(electronic signature or email or any sort of written consent)<br>[if more than one, please copy and paste this row for additional Co-Is<br><br>Do you have ethics certification? ☐ No ☒ Yes (please attach in your CV below)<br><br>Do you have RBM training certification? ☐ No ☐ Yes (please attach the certificate with CV below)	Primary Scientific Division of the Co-I<br><br>MCHD<br><br>______________________________<br><br>Approval of the Respective Senior Director/ Programme Head<br><br>(Signature)
**Co-Investigator(s) - Internal:** Sex ☒ Female ☐ Male<br><br>**Jannatul Ferdous Antu**<br><br>**Address** (Position, phone no, extension no, cell, and email address**):**<br>Senior Research Officer<br>Gender, Equity and Rights Research Group<br>Health Systems and Population Studies Division<br>Extension: 2202<br>Mobile: 01722897385<br>Email: ferdous.antu@icddrb.org<br><br>Signature or written consent of Co-I: ___________________<br>(electronic signature or email or any sort of written consent)<br>[if more than one, please copy and paste this row for additional Co-Is<br><br>Do you have ethics certification? ☐ No ☒ Yes (please attach in your CV below)<br><br>Do you have RBM training certification? ☐ No ☐ Yes (please attach the certificate with CV below)	Primary Scientific Division of the Co-I<br><br>MCHD<br><br>______________________________<br><br>Approval of the Respective Senior Director/ Programme Head<br><br>(Signature)

**Co-Investigator(s) – External:** Sex ☐ Female ☒ Male<br><br>**Dr. Md. Manjur Hossain**<br><br>**Address** (provide full official address, including land phone no(s), extension no. (if any), cell phone number, and email address):<br><br>Program Manager (A&RH), MCH-Services, DGFP<br>+8801711076553<br><br>Signature or written consent of Co-I: __ (email approval attached)________<br>(electronic signature or email or any sort of written consent)<br>[if more than one, please copy and paste this row for additional Co-Is]	
**Co-Investigator(s) – External:** Sex ☐ Female ☒ Male<br><br>**Dr. A. N. M Mosatafa Kamal Mazumder**<br><br>**Address** (provide full official address, including land phone no(s), extension no. (if any), cell phone number, and email address):<br><br>Assistant Director, Maternal & Child Health, DGFP, Ministry of Health & Family Welfare, +8801711076553<br><br>Signature or written consent of Co-I: __ (email approval attached)________<br>(electronic signature or email or any sort of written consent)<br>[if more than one, please copy and paste this row for additional Co-Is]	

Collaborating Institute(s): Please provide full official address

Institution # 1

Country	
Contact person	
Department<br>(including Division, Centre, Unit)	
Institution<br>(**with official address)	
Directorate<br>(in case of GoB i.e. DGHS)	
Ministry (in case of GoB)	

Contribution by the Members of the Scientific Team:

Members' Name	Contribution								
	Research idea/ concept	Study design	Protocol writing	Respond to external reviewers' comments	Defending at IRB	Developing data collection Tool(s)	Data Collection	Data analysis/ interpretation of results	Manuscript writing
Dr. Ruchira Tabassum Naved	☒	☒	☒	☒	☒	☒	☐	☒	☒
Mahfuz Al Mamun	☒	☒	☒	☒	☒	☒	☒	☒	☒
Aloka Talukder	☐	☒	☒	☐	☒	☒	☒	☒	☒
Sultan Mahmud	☐	☒	☒	☐	☐	☒	☒	☒	☒
Raafat Hassan	☐	☐	☐	☐	☐	☒	☒	☒	☒
Jannatul Ferdous Antu	☐	☐	☐	☐	☐	☒	☒	☒	☒

Study Population: Sex, Age, Special Group and Ethnicity

Research Subject:

☒ Human
☐ Animal
☐ Microorganism
☐ Other (specify): _____________

Sex:

☒ Male
☒ Female
☐ Transgender

Age:

☐ 0 – 4 years
☐ 5 – 10 years
☒ 11 – 17 years
☒ 18 – 64 years
☐ 65 +

Special Group:

☒ Pregnant Women
☐ Fetuses
☐ Prisoners
☐ Destitutes
☐ Service Providers
☐ Cognitively Impaired
☐ CSW
☐ Expatriates
☐ Immigrants
☐ Refugee
☐ Others (specify): ________________

Ethnicity:

☒ No ethnic selection (Bangladeshi)
☐ Bangalee
☐ Tribal group
☐ Other (specify):

NOTE: It is icddr.b's policy to include men, women, children and transgender in its research projects involving participation of humans, unless there is strong justification(s) for their exclusion.

Consent Process: (Check all that apply**)**

☐ Written
☒ Oral
☐ Audio
☐ Video
☐ None *(**if anyone of the above is checked except 'None' consent forms must be attached).*

Language:

☒ Bangla
☐ English
☐ Other (specify: _______________

a. Will study tools/questionnaire be used for this protocol?	☒ Yes ☐ No ☐ Not applicable (If yes, tools/questionnaire must be attached).

Project/Study Site: (Check all that apply**)**

- ☐ Chakaria
- ☐ Bandarban
- ☐ Dhaka Hospital
- ☐ Kamalapur Field Site/HDSS
- ☐ Mirpur (Dhaka)
- ☐ Matlab DSS Area
- ☐ Matlab non-DSS Area
- ☐ Matlab Hospital
- ☐ Mirzapur
- ☐ Bianibazar (Sylhet)
- ☐ Kanaighat (Sylhet)
- ☐ Jakigonj (Sylhet)
- ☐ Other community in Dhaka
  Name: ______________________________
- ☒ Other Name: Rajbari Sadar upazila, Rajbari
- ☐ Multi-national Study
  Name of the country____________________

Project/Study Type: (Check all that apply)

- ☐ Case Control Study
- ☐ Clinical Trial (Hospital/Clinic/Field)*
- ☒ Community-based Trial/Intervention
- ☒ Cross Sectional Survey
- ☒ Family Follow-up Study
- ☒ Longitudinal Study (cohort or follow-up)
- ☐ Meta-analysis
- ☒ Programme Evaluation
- ☐ Programme (Umbrella Project)
- ☐ Prophylactic Trial
- ☐ Record Review
- ☐ Secondary Data Analysis
  Protocol No. of Data Source:
  ____
- ☐ Surveillance/Monitoring
- ☐ Systematic Review
- ☒ Other (specify): Qualitative
  ____

***Note**: International Committee of Medical Journal Editors (ICMJE) defines Clinical Trial as "*Any research project that prospectively assigns human participants to intervention and comparison groups to study the cause-and-effect relationship between a medical intervention and a health outcome*".

PI of the RRC- and ERC-approved Clinical Trials should provide necessary information to IRB Secretariat (Research Administration) for registration and uploading into relevant websites (usually at the https://register.clinicaltrials.gov/). They should also provide relevant information to the IRB Secretariat in the event of amendment/modification after their approval by RRC and ERC.
In case of a multi-country study and if a study is registered elsewhere by the prime recipient or others; it does not need to be re-registered under icddr,b's account; provided evidence of NCT registration number is submitted to the IRB.

Biological Specimen:

a) Will the biological specimen be stored for future use?	☐ Yes ☐ No ☒ Not applicable
b) If the response is 'yes', how long the specimens will be preserved?	_________ years
c) What types of tests will be carried out with the preserved specimens?	
d) Will the consent be obtained from the study participants for use of the preserved specimen for other initiative(s) unrelated to this study, without their re-consent?	☐ Yes ☐ No ☒ Not applicable
e) Will the specimens be shipped to other country/ countries? If yes, name of institution(s) and country/countries.	☐ Yes ☐ No ☒ Not applicable<br>Name<br>______________________________
f) If shipped to another country, will the surplus/unused specimen be returned to icddr,b? If the response is 'no', then the surplus/unused specimen must be destroyed.	☐ Yes ☐ No ☒ Not applicable
g) Who will be the custodian of the specimen at icddr,b?	

h) Who will be the custodian of the specimen when shipped outside Bangladesh?	
i) Who will be the owner(s) of the specimens?	
j) Has a MoU been signed with regards to collection, storage, use and ownership of specimen?<br>If the response is 'yes', please attach a copy of the MoU.<br>If the response is 'no', appropriate justification should be provided for not signing a MoU.	☐ Yes ☐ No ☒ Not applicable

Proposed Sample Size:
Sub-group (Name of subgroup e.g. Men, Women) and Number

Name	Number	Name	Number
**Survey**		**Qualitative data collection**	
Married adolescent girls aged 15-19 years, living with husband	1120	Married adolescent girls aged 15-19 years, living with husband	16
Husbands of married adolescent girls	1120	Husbands of married adolescent girls	16
Community members aged 35-59 years	576	Key informants	6
		FGD participants	64
		**Total (including quantitative and qualitative)**	**2918**

Determination of Risk: Does the Research Involve (Check all that apply**)**

☐ Human exposure to radioactive agents?
☐ Foetal tissue or abortus?
☐ Investigational new device?
Specify: ____________________
☐ Existing data available from Co-investigator?
☐ Human exposure to infectious agents?
☐ Investigational new drug?
☐ Existing data available via public archives/sources?
☐ Pathological or diagnostic clinical specimen only?
☐ Observation of public behaviour?
☐ New treatment regime?

	Yes	No
Will the information be recorded in such a manner that study participants can be identified from the information directly or through identifiers linked to the study participants?	☒	☐
Does the research deal with sensitive aspects of the study participants' sexual behaviour, alcohol use or illegal conduct such as drug use?	☒	☐

Could information on study participants, if available to people outside of the research team:

	Yes	No
a) Place them at risk of criminal or civil liability?	☐	☒
b) Damage their financial standing, reputation or employability, or social rejection, or lead to stigma, divorce etc.?	☐	☒

Do you consider this research: (check one)

☒ Greater than minimal risk	☐ No more than minimal risk	☐ Only part of the diagnostic test

Note: Minimal Risk: The probability and the magnitude of the anticipated harm or discomfort to participants is not greater than those ordinarily encountered in daily life or during the performance of routine physical, psychological examinations or tests, e.g. the risk of drawing a small amount of blood from a healthy individual for research purposes is no greater than when the same is performed for routine management of patients.

**Risk Group of Infectious Agent and Use of Recombinant DNA**	
a) Will specimens containing infectious agent be collected?	☐ Yes ☐ No ☒ Not applicable
b) Will the study involve amplification by culture of infectious agents?	☐ Yes ☐ No ☒ Not applicable
c) If response to questions (a) and/or (b) is 'yes', to which Risk Group (RG) does the agent(s) belong? (Please visit http://shetu.icddrb.org/index.php?option=com_content&view=article&id=265&Itemid=677 to review list of microorganism by Risk Group)	☐ RG1 ☐ RG2 ☐ RG3 ☐ RG4
d) Does the study involve experiments with recombinant DNA?	☐ Yes ☐ No ☒ Not applicable

Does the study involve any biohazards materials/agents or microorganisms of risk group 2, 3, or 4 (GR2, GR-3 or GR4)?

☐ Yes ☒ No

[If the response is 'yes'] I, (print name of the PI) affirm that we will use the standard icddr,b laboratory procedures for biosafety of the hazardous materials/agents or microorganisms in the conduction of the study.

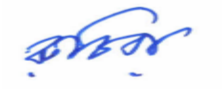

Signature of the Principal Investigator **Date : 17. 11.2022**

Dissemination Plan: [please explicitly describe the plans for dissemination, including how the research findings would be shared with stakeholders, identifying them if known, and the mechanism to be used; anticipated type of publication (working papers, internal (institutional) publication, international publications, international conferences/seminars/workshops/ agencies. [Check all that are applicable]

**Dissemination type**	**Response**		**Description (if the response is a yes)**
Seminar for icddr,b scientists/ staff	☐ No	☒ Yes	
Internal publication	☒ No	☐ Yes	
Working paper	☒ No	☐ Yes	
Sharing with GoB (e.g. DGHS/ Ministry, others)	☐ No	☒ Yes	
Sharing with national NGOs	☐ No	☒ Yes	
Presentation at national workshop/ seminar	☐ No	☒ Yes	
Presentation at international workshop/ conference	☐ No	☒ Yes	
Peer-reviewed publication	☐ No	☒ Yes	
Sharing with international agencies	☐ No	☒ Yes	
Sharing with donors	☐ No	☒ Yes	
Policy brief	☐ No	☒ Yes	
Other			
Other			

Funding:

Is the protocol fully funded?	☒ Yes	☐ No
If the answer is yes, please provide sponsor(s)'s name	1. Department of Foreign Affairs, Trade and Development (DFATD)	
	2.	
Is the protocol partially funded?	☐ Yes	☐ No
If the answer is yes, please provide sponsor(s)'s name	1.	
	2.	

If fund has not been identified:

Is the proposal being submitted for funding?	☐ Yes	☐ No
If yes, name of the funding agency	1.	
	2.	

Conflict of interest:
Do any of the participating investigators and/or member(s) of their immediate families have an equity relationship (e.g. stockholder) with the sponsor of the project or manufacturer and/or owner of the test product or device to be studied or serve as a consultant to any of the above?

☒ No ☐ Yes (please submit a written statement of disclosure to the Executive Director, icddr,b)

Proposed Budget:

Dates of Proposed Period of Support Period ($)

(Day, Month, Year - DD/MM/YY)

Beginning Date : 01 January, 2023

End Date : 31 March, 2025

Cost Required for the Budget

Years	Direct Cost	Indirect Cost	Total Cost
**Year-1**	163,034	23,444	186,479
**Year-2**	159,031	22,869	181,899
**Year-3**			
**Year-4**			
**Total**	**322,065**	**46,313**	**368,378**

Has the protocol been presented amongst Division level research forum for their inputs/comments and advice/ suggestions? Whether the comments received from the research forum are addressed in the protocol?

☒ Yes ☐ No

If 'no' is the response, its reason(s) in brief:

Certification by the Principal Investigator:

I certify that the statements herein are true, complete and accurate to the best of my knowledge. I am aware that any false, fictitious, or fraudulent statements or claims may subject me to criminal, civil, or administrative penalties. I agree to accept the responsibility for the scientific conduct of the project and to provide the required progress reports including updating protocol information in the NAVISION if a grant is awarded as a result of this application.

I also certify that I have read icddr,b Data Policies and understand the PIs' responsibilities related to archival and sharing of research data, and will remain fully compliant to the Policies. (Note: The Data Policies can be found here: http://shetu.icddrb.org/index.php?option=com_content&view=article&id=273&Itemid=685)

Signature of PI **Date:** 17.11.22

Approval of the Project by the Division Director of the Applicant:

The above-mentioned project has been discussed and reviewed at the Division level.

Dr. S.M. Manzoor Ahmed Hanifi
Name of the Division Director

Signature

20/11/2022
Date of Approval

Table of Content

RRC APPLICATION FORM 1
List of Abbreviations... 16
Project Summary 17
Description of the Research Project 20
Hypothesis to be tested:.. 20
Specific Objectives:.. 20
Background of the Project including Preliminary Observations: 20
Research Design and Methods 24
Sample Size Calculation and Outcome (Primary and Secondary) Variable(s)............................ 30
Data Analysis 36
Data Safety Monitoring Plan (DSMP).. 37
Ethical Assurance for Protection of Human rights.. 38
Use of Animals 40
Collaborative Arrangements.. 40
Facilities Available.. 40
Literature Cited.. 40
Budget... 46
Biography of the Investigators .... **Error! Bookmark not defined.**
Consent form (ENGLISH and BANGLA)...47
Check-List .... **Error! Bookmark not defined.**
Annex A. Survey questionnaire and qualitative guides, both English and Bangla**Error! Bookmark not defined.**
Annex B. Gender Analysis Tool 48
Annex C. Responses to external reviewers' comments .... **Error! Bookmark not defined.**
Annex D. Ethics certificates for all researchers .... **Error! Bookmark not defined.**

☒ Check here if appendix is included

List of Abbreviations

SRHR - Sexual and reproductive health and rights

SRH - Sexual and reproductive health

FP - Family planning

DV - Domestic violence

BHDSS - Baliakandi health and demographic surveillance system

KII - Key Informant Interview

IDI - In-depth Interview

FGD – Focus group discussion

Project Summary
[The summary, within a word limit of 300, should be stand alone and be fully understandable.]

Principal Investigator: Ruchira Tabassum Naved, PhD
Research Protocol Title: Development and testing of Balika Bodhu: a social norm intervention to address denial of sexual and reproductive health and rights to married adolescent girls in rural Bangladesh
Proposed start date: 01/01/2023 Estimated end date: 31/03/2025

Background (brief):

(i) Burden:

Sexual and reproductive health and rights (SRHR) are vital to one's health and development. Over the last few decades Bangladesh, known as a patriarchal society, has made remarkable progress in improving contraceptive use, and maternal health. However, the progress has not been consistent in different SRHR domains and is not uniform across different groups of reproductive aged females. The progress was particularly slow in realising SRH rights of the women. Evidence suggests that choice and consent of females are ignored in all spheres of SRHR and that patriarchal gender and social norms, and discriminatory laws and practices in Bangladesh perpetuate denial of SRHR to females. Due to high prevalence of child marriage in Bangladesh (59%) (NIPORT & ICF, 2019) a huge proportion of adolescent girls are married before reaching 18. In a patriarchal setting such as Bangladesh with very strong gender and age hierarchies these child brides typically command the lowest power in the marital home. They typically have lower education than those who get married in adulthood. They also lack voice and bargaining skills. In such a context, the husband or mother-in-law may figure prominently in decisions about using contraception and the timing of childbearing. In this context, denial of SRHR usually becomes part of their lived experiences.

(ii) Knowledge gap:

Although social norms are recognised as one of the main drivers of denial of SRHR to married adolescent girls (MAG), social norms theories and approaches to programming are relatively new and there has been little attempt to develop and rigorously test interventions to address such social norms worldwide and in Bangladesh. Also, a handful of programmes have been implemented worldwide and in Bangladesh to empower partnered female adolescents for promoting SRHR. But, we argue that conceptualization of empowerment in these initiatives were often not grounded in solid theory and evidence. Thus, empowerment programmes for women and girls often fail to include a critical component, namely, movement building and collective agency and critical consciousness, without which empowerment cannot take place in its true sense.

(iii) Relevance:

The current study proposes to develop and test Balika Bodhu, a social norm intervention to address the gaps in programming and in the literature combining a comprehensive empowerment programme for MAGs with engagement of other stakeholders to increase demand, access and practice of positive behaviours in relation to SRHR among these MAGs. Balika Bodhu covers married adolescent girls aged 15-19 years.

Hypothesis (if any):

Balika Bodhu, a social norm intervention on sexual and reproductive health and rights (SRHR) combining a comprehensive empowerment package for married adolescent girls (MAGs) and positive engagement of relevant stakeholders will significantly improve demand, access and practices regarding SRHR among MAGs in family planning (FP) use, conception, and SRH service uptake.

Objectives:

Primary objective:

(iv) To develop Balika Bodhu, a social norm intervention combining empowerment of MAGs and mobilisation of relevant stakeholders to promote demand for, access to, and practices of SRHR, including FP use, conception and SRH service uptake.

(v) To measure the effect of Balika Bodhu in increasing demand for, access to, and practices of SRHR (FP use, conception, and SRH service uptake) of MAGS.

Secondary objectives:
(vi) To measure the effect of Balika Bodhu on empowerment of MAGs;
(vii) To measure the effect of Balika Bodhu on social norm regarding SRHR of MAGs;
(viii) To explore how change happens or why change does not occur.

Methods:

Balika Bodhu will involve a mixed-method two-arm (intervention and control) Cluster Randomized Controlled Trial (CRCT) design. Balika Bodhu will be implemented in 32 clusters (villages, primary sampling unit) in Rajbari Sadar *upazila* of Rajbari district. The clusters will be formed and randomized to two study arms. The study will employ both quantitative and qualitative methods to test the impct of Balika Bodhu to promote demand for, access to and practices of SRHR, including FP use, conception and and SRH service uptake. A total of 1120 MAGs will be randomly selected (35 per cluster) from the list obtained through household enumeration. Their husbands will also be included in the study. Both the MAGs and their husbands will be surveyed at baseline, will be provided with the intervention in the intervention clusters and will be surveyed at endline. Randomly selected cross sectional samples of community members aged 35-59 years will also be surveyed at baseline and endline. Qualitative data will be collected at baseline and endline from two intervention clusters using 32 In-depth Interviews (with 16 MAGs and 16 husbands of MAGs), and 6 Key Informant Interviews (with mothers-in-law of MAGs, NGO staff, CHWs, village doctors/ traditional healers/ drug sellers) and 8 Focus Group Discussions (with MAGs, their spouses, and elderly women/influential community members aged 35-59 years).

Outcome measures/variables:

Primary outcomes:

1. Instrumental agency of MAGs in decision making regarding SRHR (i.e., FP use; timing of first conception; and number of children to have)

Secondary outcomes:

Secondary outcomes that will be measured among the MAGs:

2. Intrinsic agency of MAGs
3. Instrumental agency of MAGs
4. Collective agency of MAGs
5. Critical consciousness (perceived inequality, egalitarianism and critical action) regarding gender inequality in the society
6. Knowledge of MAGs regarding FP and conception
7. Positive attitudes of MAGs regarding FP and conception
8. Couple communication initiated by MAGs regarding FP use, timing of first conception, and number of children to have
9. MAGs attempts to negotiate FP use, timing of first conception, and number of children to have as per her choice
10. Service uptake among MAGs regarding abortion and post-abortion care, and help seeking after experiencing violence
11. Prevalence of intimate partner violence (IPV) among the MAGs

Secondary outcomes that will be measured among the husbands of MAGs:

12. Knowledge of husbands of MAGs regarding FP and conception
13. Positive attitudes of husbands of MAGs regarding FP and conception
14. Positive attitudes regarding agency of MAGs regarding SRHR among the husbands
15. Non-condoning attitudes of husbands of MAGs regarding violence against women
16. Positive attitudes of husbands of MAGs regarding gender roles within household

Secondary outcomes that will be measured among the community members:

17. Knowledge of community members regarding FP and conception
18. Positive attitudes of community members regarding FP and conception
19. Positive attitudes regarding agency of MAGs regarding SRHR among the community members
20. Positive attitudes of community members regarding violence against women
21. Positive attitudes of community members regarding gender roles within household

<u>Secondary outcomes that will be measured among both the MAGgs and the community members:</u>

22. Positive social norms regarding SRHR of MAGs

Description of the Research Project

Hypothesis to be tested:

In a hypothesis testing research proposal, briefly mention the hypothesis to be tested and provide the scientific basis of the hypothesis, critically examining the observations leading to the formulation of the hypothesis.

Does this research proposal involve testing of hypothesis: ☐ No ☒ Yes (describe below)
Hypotheses:

Balika Bodhu, a social norm intervention on sexual and reproductive health and rights (SRHR) combining a comprehensive empowerment package for married adolescent girls (MAGs) and positive engagement of relevant stakeholders will significantly improve demand, access and practices regarding SRHR among MAGs in FP use, conception and SRH service uptake (See Figure 1 and pages 21-22 for details).

Specific Objectives:

Describe the specific objectives of the proposed study. State the specific parameters, gender aspects, biological functions, rates, and processes that will be assessed by specific methods.

Primary objective:

(1) To develop Balika Bodhu, a social norm intervention combining empowerment of MAGs and mobilisation of relevant stakeholders to promote demand for, access to, and practices of SRHR, including family planning (FP) use, conception, and SRH service uptake.
(2) To measure the effect of Balika Bodhu in increasing demand for, access to and practices of SRHR (FP use, conception, and SRH service uptake) of MAGS.

Secondary objectives:

(3) To measure the effect of Balika Bodhu on empowerment of MAGs;
(4) To measure the effect of Balika Bodhu on social norm regarding SRHR of MAGs;
(5) To explore how change happens or why change does not occur.

Background of the Project including Preliminary Observations:

Provide scientific validity of the hypothesis based on background information of the proposed study and discuss previous works on the research topic, including information on sex, gender and diversity (ethnicity, SES) by citing specific references. Critically analyze available knowledge and discuss the questions and gaps in the knowledge that need to be filled to achieve the proposed aims. If there is no sufficient information on the subject, indicate the need to develop new knowledge.

Sexual and reproductive health and rights (SRHR) are vital to one's health and development. It is recognised as essential for achieving social justice (Cepal, 2013). According to the Guttmacher–Lancet Commission, achieving SRH rests on realizing sexual and reproductive rights including the right to control one's own body, define one's sexuality, choose one's partner, and receive confidential, respectful, and high-quality health services (Starre, 2018). However, denial of SRH rights and limited access to the full set of SRHR services to females have been pervasive in many countries and particularly in countries with patriarchal social structures, where female power and status are compromised.

Over the last few decades Bangladesh, known as a patriarchal society, has made remarkable progress in improving contraceptive use, and maternal health (NIPORT & ICF, 2019; MoHFW, et al., 2015). However, the progress has not been consistent in different SRHR domains and is not uniform across different groups of reproductive aged females (UNFPA, 2016). Evidence suggests progress was particularly slow in realising SRH rights of the women (Naripokkho, 2017; BLAST, 2022) suggests that choice and consent of females are ignored in all spheres of SRHR and that patriarchal gender and social norms, and

discriminatory laws and practices in Bangladesh perpetuate denial of SRHR to females (Naripokkho, 2017; BLAST, 2022).

Marriage is almost universal in Bangladesh (Islam and Mahmud, 1996). Due to high prevalence of child marriage in Bangladesh (59%) (NIPORT & ICF, 2019) a huge proportion of adolescent girls are married before reaching 18. Since marriages are mostly arranged and village exogamy is largely practiced, most girls are cut off from their own networks as they join their marital family usually living in a different village. In a patriarchal setting such as Bangladesh with very strong gender and age hierarchies these child brides typically command the lowest power in the marital home. They typically have lower education than those who get married in adulthood. They also lack voice and bargaining skills (Gage, 2000; MacQuarrie, 2009; Shahabuddin et al., 2016). In such a context, the husband or mother-in-law may figure prominently in decisions about using contraception and the timing of childbearing (Barua and Kurz 2001; MacQuarrie and Edmeades, 2015) or she may begin childbearing as a way to improve her standing in the household (MacQuarrie 2009; MacQuarrie 2015). In this context, denial of SRHR usually becomes part of their lived experiences. For instance, they are commonly denied consent and choice regarding when to conceive, how many children to have, use of FP, and SRH care seeking (Shahabuddin et al., 2016; Shahabuddin et al., 2016; Samandari et al., 2020; Haque et al., 2012; Deb et al., 2011). They end up having teenage pregnancy, shorter birth intervals and higher number of children (Godha et al., 2013; Santhya & Jejeebhoy, 2015; MacQuarrie KLD et al., 2015).

Although social norms are recognised as one of the main drivers of denial of SRHR to married adolescent girls (Coast et al., 2019; Shakya et al., 2019; Wamoyi et al., 2019), social norms theories and approaches to programming are relatively new and there has been little attempt to develop and rigorously test interventions to address such social norms worldwide. In line with other proponnets of social norm interventions we argue that in contexts, where norms are significant drivers of decision-making and behaviour, social norm interventions are most likely to be effective in addressing the issues sustainably (Gonzales et al., 2012; Igras et al., 2019). A handful of social norm-based interventions have been developed for addressing adolescent SRHR in LMIC. Most of them are focused on child marriage (Naved et al., 2022), violence (Clark et al., 2017) and female genital mutilation (Cisse et al., 2018). A very few social norm interventions addressing other SRHR issues faced by married adolescent girls have been rigorously evaluated. The two qualitative evaluations deserving mentioning are of CARE projects known as Tesfa (Addis Continental Institute of Public Health, 2018) and Abdiboru (Addis Continental Institute of Public Health, 2016) interventions in Africa. These projects aimed at promoting ASRHR knowledge, positive attitudes and uptake of SRH services empowering the MAGs and working with the communities they live in. One of the most recent social norm interventions addressing ASRHR of married girls was the IMAGINE project of CARE implemented in Bangladesh (CARE, 2019; Grant and Laterra, 2019). Evaluation of all of these CARE projects show some promise. However, none of them were rigorously evaluated.

A lot of programmes have been implemented worldwide and in Bangladesh to empower partnered female adolescents for promoting SRHR. We argue that conceptualization of empowerment in these initiatives were often not grounded in solid theory and evidence. Thus, empowerment programmes for women and girls often fail to include a critical component, namely, movement building and collective agency and critical consciousness, without which empowerment cannot take place in its true sense (Freire, 1973; Yount et al., 2021). Following feminist traditions, we define empowerment as intrinsic power within to overcome dominated consciousness, instrumental power to pursue aspirations, and collective power with others to pursue shared goals (Thomas et al., 2021; Sardenberg, 2008). We are going to address these research and intervention gaps in this project.

At this backdrop, the current study proposes to develop and test Balika Bodhu, a social norm intervention to address the gaps in programming and in the literature combining a comprehensive empowerment programme for MAGs with engagement of other stakeholders to increase demand, access and practice of positive behaviours in relation to SRHR among these MAGs. Balika Bodhu covers married adolescent girls aged 15-19 years.

The Balika Bodhu Theory of Change (ToC)

Figure 1 depicts the Theory of Change (ToC) that the Balika Bodhu intervention is guided by. The ToC has been adapted from the Tipping Point initiative's Gender Empowerment and Women's Voice Framework[1]. Empowerment of MAGs can be defined as the expansion of capabilities to participate in, negotiate with, influence, control, and hold accountable the institutions that affect their lives. The intervention will focus on three domains of change, namely (1) individual agency that builds awareness and empowers MAGs with knowledge, skills and capabilities about equality, rights, SRHR, livelihood options, and social norms. Individual agency is manifested in women's aspirations, resources, actions and achievements; (2) power relations through which MAGs navigate their lives (e.g., negotiating their SRH needs and rights with other social actors, including men; and taking greater charge of their body/sexuality); and (3) the structures that support demand, access and domains of SRHR of MAGs. Structures include routines, patterns of relationships and interaction, and conventions that lead to taken-for granted behavior; institutions that establish agreed-upon meanings, accepted ("normal") forms of domination (who "naturally" has power over what or whom), and agreed criteria for legitimizing the social order. These three domains are intimately related, structuring and influencing one another. Both agency and structure are mediated through relationships between and among social actors while, at the same time, forms and patterns of relationships are deeply influenced – frequently in hidden ways – by agency and structure. Empowerment, in part, consists in individual women building relationships, joint efforts, coalitions, and mutual support, in order to claim and expand agency, alter inequitable structures, and so realize rights and livelihood security.

The inputs (on the far left of Figure 1) illustrate the project components, which will impact each of these domains in turn. At the individual level, through group sessions and activities, the intervention will build agency and assets of MAGs, and will change power relations in the family, and in the community. Relationally, the intervention will target the spouses of MAGs, community members to encourage more trusting relationships with the MAGs that support their demand, access and practices of SRHR and work in solidarity to achieve those. Together, these changes will contribute to improve demand, access and practices of SRHR of MAGs.

[1] https://caretippingpoint.org/theory-of-change-2-2/#:~:text=The%20Tipping%20Point%20ToC%20underlines,critical%20consciousness%20of%20one's%20rights.

Figure 1. Balika Bodhu Theory of Change (ToC)

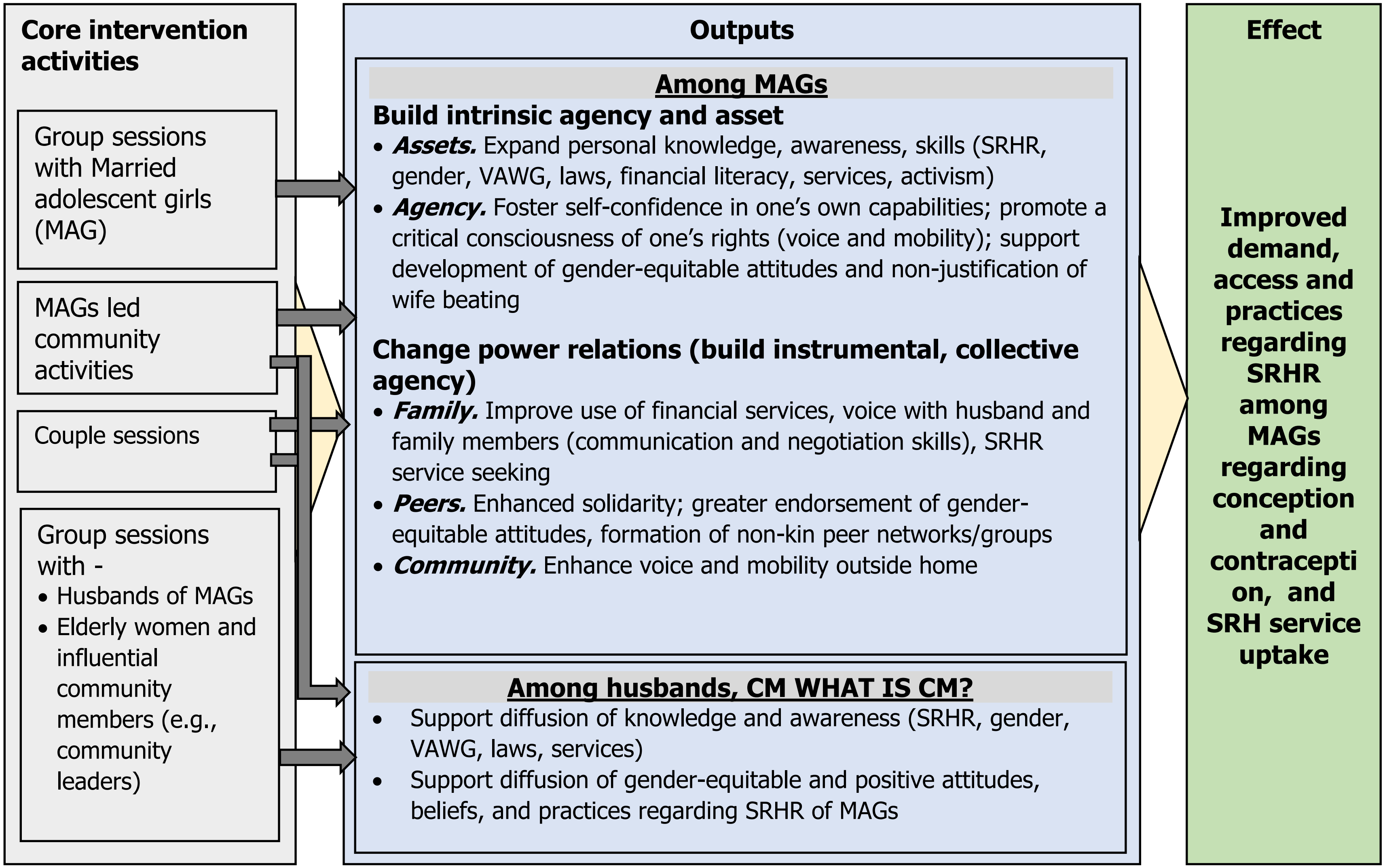

The Social Norm intervention

In line with the ToC the Balika Bodhu intervention will be developed heavily drawing on CARE's IMAGINE model (CARE, 2019), and on SAFE, a project spearheaded by icddr,b and aimed at promoting SRHR and addressing violence against women in slums of Dhaka (Naved et al., 2018).

Balika Bodhu intervention will be implemented by Nari Maitree, an NGO working on women's issues. The research team will conduct both quantitative and qualitative monitoring for documenting implementation fidelity. This will allow tracking of any deviations from the charted programme, which will be important to take into consideration during interpretation of the results. Effectiveness of the intervention will be assessed in changing social norms endorsing denial of SRHR to MAGS and in empowering them to demand, access and practice SRHR. The intervention will involve a 12-month gender transformative participatory programming for: (1) empowering MAGs and (2) creating an enabling environment in the family and the community for the MAGs to demand, access and practice SRHR. For empowering MAGs their intrinsic, instrumental and collective agency will be promoted. Special attention will be paid to incorporate two critical elements, often missing from many empowerment programmes – development of critical consciousness and movement building. The intervention will particularly focus on promoting rights of the MAGs through enhancement and exercise of the notions of choice and consent.

The intervention will target Married adolescent girls, their spouses, and elderly/influential community members. Thirty-six 2-hour weekly sessions will be conducted with MAGs. The session topics will be categorized into two broad headings: i) Health and life skills sessions covering, for example topics such as sex and gender, rights, marriages, SRHR (e.g., first conception, birth spacing, contraception, healthy relationships, polygamy, divorce, gender-based violence (GBV), communication and negotiation, anger and conflict management, goal setting, household budgeting, activism, leadership skills, and community action planning, and ii) Business and entrepreneurship sessions, which will basically include sessions smart planning of businesses (e.g., market research, business planning, and adding value).

Spouses of the MAGs will receive 20 2-hour bi-weekly sessions. The session topics will cover: sex and gender, rights, marriages, registration, dowry, conception and contraception, healthy relationships, polygamy, divorce, gender-based violence (GBV), communication and negotiation, anger and conflict management, goal setting, and household budgeting.

A total of three 2-hour couples sessions will be held, each to be delivered every two months, starting from the 6th month of the intervention. The session topics will include teenage pregnancy, unwanted pregnancy, and action planning. Elderly and influential community members will have three 2-hour bi-monthly sessions starting from the 6th month of the intervention, covering topics related to teenage pregnancy, unwanted pregnancy, and action planning.

MAGs with leadership qualities will be identified and trained to conduct campaigns and activism. They will be put in contact with other girls' groups and networks, mentored and given access to resources to execute at least two community-level social norm activities. These leaders will also receive training on income generating.

Monitoring of Balika Bodhu

We propose routine quantitative monitoring and reporting of number of group sessions by schedule, duration of group session, number of session attendants, number of activities performed, etc. We will also keep record of how many sessions and which particular sessions the participants are attending. This will allow us to measure the impact of number of session attendance on the primary and secondary outcomes. It is preferable to conduct such reporting bi-weekly so that problems can be identified and fixed before it is too late. Presentation of performance of each cluster graphically will allow all staff to understand their own performance in relation to others' and may encourage catching up.

Monitoring qualitative aspects of the intervention is of utmost importance. We will conduct three rounds of qualitative monitoring as follows:

Round	Timing	Data collection techniques and mumbers
Round 1	3rd month	• <u>Session observation</u>: 5 sessions with MAGs, and 3 with husbands

		• Short interviews with the group members: 5 with MAGs and 3 with husbands<br>• FGDs with the group session facilitators: 1 with session facilitators and 1 with co-facilitators
Round 2	6th month	• Session observation: 5 sessions with MAGs, and 3 with husbands, 2 with couples, and 2 with community members<br>• Short interviews with the group members: 5 with MAGs and 3 with husbands<br>• FGDs with the group session facilitators: 1 with session facilitators and 1 with co-facilitators
Round 3	9th month	• Session observation: 5 sessions with MAGs, and 3 with husbands, 2 with couples, and 2 with community members<br>• Short interviews with the group members: 5 with MAGs and 3 with husbands<br>• FGDs with the group session facilitators: 1 with session facilitators and 1 with co-facilitators<br>• Observation of community event: 3

Instant feedback to the facilitators after session observation will help the facilitators in understanding their weaknesses and strengths and create an opportunity to improve performance. Observation reports will also be shared at higher levels making these levels aware of the weaknesses and strengths of the facilitators and the challenges they face. This will enable timely support to the facilitators. FGDs with the facilitators will help in identifying the challenges they face and in coming up with solutions collectively. Short interviews with the group members will enable us to understand the challenges the group members face in attending, understanding and participating in the sessions.

Research Design and Methods

Describe the research design and methods and procedures to be used in achieving the specific aims of the research project. If applicable, mention the type of personal protective equipment (PPE), use of aerosol confinement, and the need for the use BSL2 or BSL3 laboratory for different part of the intended research in the methods. Define the study population with inclusion and exclusion criteria, the sampling design, list the important outcome and exposure variables, describe the data collection methods/tools, and include any follow-up plans if applicable. Justify the scientific validity of the methodological approach (biomedical, social, gender, or environmental).

Also, discuss the limitations and difficulties of the proposed procedures and sufficiently justify the use of them.

Definitions

In the current study we use the following operational definitions.

Social norms are context-dependent, externally-derived rules of obligatory, appropriate, and acceptable behaviour shared by people in the same group or society (Bell & Cox, 2015; Cislaghi & Heise, 2018; Mackie et al., 2015).

Women's empowerment is multidimensional (Agarwala & Lynch, 2006; Malhotra & Schuler, 2005; Mason, 2005), capturing the process by which women claim enabling resources to enhance their agency, or ability to make strategic choices under constraints (Kabeer, 1999; Kabeer, 2005).

Agency, itself, is a multidimensional construct that involves internal states of being, ways of acting, and ways of being and acting jointly with others (Kabeer, 1999).

Intrinsic agency or *power within*—is analogous to the concept of thin relational autonomy (Khader, 2016). It entails a critical awareness of one's rights and aspirations, confidence in one's capabilities, and motivation to pursue self-defined goals.

Instrumental agency involves the creative power to exercise one's capabilities; make one's own strategic choices; pursue one's rights, goals, and aspirations; and affect desired change in one's life (Yount et al., 2016; Kishore & Gupta, 2004; Yount, 2005). Instrumental agency may be enacted, for example, by

expressing views that oppose prevailing norms and power relations; influencing household decisions; and moving freely in public spaces that historically have been reserved for men.

Collective agency involves engagement in or leadership of groups or networks with shared goals; confidence in the group's or network's ability to act on shared goals, and influential joint actions in pursuit of shared goals (Kabeer, 1999; Eger et al., 2018; Miedema et al., 2018; Bandura, 2000; Fukuda-Parr, 2003.

Critical consciousness, is defined as levels of consciousness and action that produce potential for change at one or more socio-ecosystemic (e.g., individual, institutional) levels (Jemal, 2016). A person with a high level of transformative potential critically reflects on the conditions that shape his or her life and actively works with self and/ or others to change problematic conditions (Campbell and MacPhail, 2002; Jemal, 2016). The process of transformation requires the simultaneous and reciprocating processes of objectifying and acting (Freire, 1973). One cannot truly perceive the depth of the problem without being involved in some form of action confronting the problem (Corcoran et al., 2015; Freire, 1973).

Study design

The Balika Bodhu evaluation trial employs a mixed-method design, including quantitative and qualitative components. It includes a two-arm Cluster Randomized Controlled Trial (CRCT) design. The arms are as follows:

Arm 1: The social norm intervention
Arm 2: Pure control

Study site

Balika Bodhu will be implemented in Rajbari Sadar upazila of Rajbari district has been selected as the study site for the following reasons: (1) the rate of child marriage in Rajbari district is 56%, which is close to the national rate of 59% (BBS and UNICEF, 2019; NIPORT and ICF, 2019); (2) It includes a large number of villages necessary for accommodating the required number of clusters (32) with buffer zones for averting any intervention contamination; (3) The villages here have adequate number of MAGs for achieving the required cluster size (i.e., 35 or more) for forming an MAG group with at least 25 members per village.

Cluster formation and randomization,

The villages from Rajbari Sadar upazila will be treated as clusters or the primary sampling units in this study. The list of villages and the number of households in each village will be obtained from the upazila statistics office. Any village/cluster with approximately 500 or more households will be considered eligible to be included in the study for allowing an adequate number of MAGs. Three teams, each consisting of two members, will visit each of the eligible villages, talk to local people, check which could be selected as study clusters and which could be potential buffers. All the teams will then finalize the list of eligible villages after ensuring sufficient buffer zone.

We will then randomly select 32 clusters/villages from the list of eligible villages. The selected 32 clusters will be subsequently assigned randomly to either the intervention or control group in a 1:1 ratio. The randomization process will be carried out by an independent statistician from icddr,b who will not be involved in the designing the study, recruitmenting the study participants, implementing the intervention or analysing the data.

Household enumeration, *sampling and recruitment of study participants*

Once the clusters have been selected and randomized to intervention and control arms, an enumeration of the households in each cluster will be conducted to collect socio-demographic information on the household members (e.g., sex, date of birth, age and marital status) to form the sampling frame for the MAGs', their husbands' and the community survey. We will also collect information on whether the MAGs living with husband or not for determining whether or not they are eligible for participating in the intervention. A properly trained team of data collectors will conduct this enumeration. Special care will be taken to train them on how to collect and check information on age. The MAGs aged between 15 and 19 years, currently living with husband and living in the study village will be considered as eligiable to participate in this study. Adult male and female community members who are aged 35-59 years and currently living in the study village will be considered as eligiable.

From the list of eligible MAGs in a cluster, we will randomly select 35 MAGs using a computer-generated sequence, once again by the same independent statistician. The number 35 was calculated assuming a 10% non-response in the baseline survey and another 20% of the MAGs/husbands of MAGs may refuse to participate in the intervention, which will make the MAG and husband of MAG groups size 25 for the group sessions in each cluster. If any cluster consists of less than 35 MAGs, one portion of the most adjacent village will be included as a part of the cluster to make the number of MAGs 35. The community survey sample will also be selected randomly using a computer-generated sequence by the same statistician.

Upon receiving consent, the selected MAGs and their husbands will be enrolled in the study and included in the baseline survey. Following the baseline survey interviews, MAGs from the intervention villages will be invited to participate in the Balika Bodhu intervention, and upon receiving their consent, they will be formally enrolled in the intervention. All MAGs and their husbands who were interviewed at baseline will also be targeted for follow-up interviews at the endline.

For the community survey, separate cross-sectional samples will be drawn and interviewed at baseline and endline. We will obtain lists of eligible community members (females and males), aged 35-59 years from household enumeration. Nine females and nine males will be randomly selected from each cluster for inclusion in the surveys making the total in each round 576.

Blinding
No one will be blinded during this selection and allocation process in this study.

Quantitative component

Study Population
The study population will be married adolescent girls (MAGs) aged 15-19 years, their husbands, and community members aged 35-59 years.

Inclusion and exclusion criteria for clusters
Any village with around 500 households or 35 MAGs will be considered as eligible to be included in this study.

Inclusion and exclusion criteria for individual participants

<u>Inclusion criteria</u>
Criteria for inclusion of the MAGs in the study will be: i) adolescent girls aged 15-19 years; and ii) currently married and living with husband; and iii) both the girl and her husband have no intention of migrating out within the next 15 months. The husbands of the selected adolescent girls will also be included in the study. Community members aged 35-59 years will also be considered eligible for participating in the study.

<u>Exclusion criteria</u>
MAGs who are physically or mentally unstable to participate in the intervention and to respond to the interview questions will be excluded from the study. Any couple having plans to migrate out during the next one and a half years will not be included in this study.

Survey tools
Three separate questionnaires will be developed to interview the married adolescent girls, their husbands, and the community members. The MAGs will be asked about their socio-economic and demographic characteristics, intrinsic, instrumental and collective agency, FP, first conception, unwanted pregnancy, birth interval, intimate partner violence (IPV), etc. In each of the SRHR domains studied, we will explore adolescent girls’ knowledge, attitudes and practices with a focus on communication, negotiation, consent, choice and decision-making. Husbands of the MAGs will be asked about their knowledge and attitudes regarding SRHR relevant to them and to the MAGs, while the community members will be asked about social norms regarding SRHR of MAGs. The questionnaires will be developed in English and then translated into Bengali. The Bengali version of the questionnaires will be pre-tested, piloted and finalized. The English version of the questionnaires will be updated following the final Bengali questionnaire.

Data collection and management

Data will be collected at baseline and endline through face-to-face interviews conducted by gender-matched interviewers upon receiving consent from the study participants. The data will be recorded using Tablets. An offline-based survey software will be developed for the purpose. Interviews will be conducted in private in a location convenient for the participants.

A survey team of six female and six male data collectors, two supervisors, one programmer and one survey coordinator will be employed in the survey. The survey team will be thoroughly trained not only in survey methods, the questionnaire, use of tablets and field procedures, but also in gender and rights, empowerment, gender-based violence, and ethics of conducting research on sensitive issues. The total training including the piloting will take around 12 days. The training will be participatory in nature.

Survey data quality monitoring
A strong data quality monitoring system will be in place. The supervisor will observe the quality of the interviews, keep notes and discuss problems during daily review sessions. The interviewers will cross-check each other's interviews randomly and provide feedback to the interviewers. The survey coordinator will randomly choose interviewers for spot checks. The data collected will be uploaded on a designated server at the end of each business day, which will allow the researchers to instantly review the data from the server. Further, a software-based data checking routine will be developed which will allow identifying inconsistencies in the data within a short period of time. A researcher will analyse the data on a weekly basis to identify any inconsistencies in the data and provide feedback to the survey team. Correction will be performed following standard procedure. Five percent of the study participants will be revisited by the supervisors and the coordinator for administering a short questionnaire as part of quality check. In addition, the researchers will visit the field on a regular basis, observe interviews, cross-check interviews, and provide feedback.

Qualitative Component
This component will basically help us to compliment the quantitative component. If change happens as a result of the intervention, the qualitative study will provide insight into how change happens through empowerment of MAGs and social norm change. If change does not take place, it would help us understand what went wrong.

Study population, sampling, recruitment in the cohort,

The qualitative study will cover two intervention clusters. Data will be collected through Key Informant Interviews (KIIs), In-depth Interviews (IDIs), and Focus Group Discussion (FGDs). While KIIs and FGDs will provide data on social norms and general practices around SRHR of MAGs, IDIs will give us access to in-depth data on individual perceptions, practices and experiences.

We will conduct **KIIs** with mothers-in-law (MiLs) of the MAGs, Non-government Organization (NGO) staff implementing SRHR programmes in the study area, Community Health Workers (CHWs), village doctors / traditional healers /drug sellers. We propose to conduct KIIS with MiLs of MAGs since they are likely to be knowledgeable about the SRHR of the MAGs. NGO staff implementing SRHR programmes addressing SRHR due to their work experiences and interpersonal skills — make them credible and knowledgeable source of information for this research. We assume that MAGs may seek support and services for different SRHR issues from the traditional healers, village doctors/drug sellers, and NGO workers, making them important source of information.

We will conduct **IDIs** with the MAGs and their spouses. Criteria for inclusion of the respected respondents for IDIs are given below:

a) *Married Adolescent Girls*
Criteria for inclusion in this cohort to be females: i) aged 15-19 years; ii) currently married and living with husband; and iii) currently none of the spouses are planning to migrate out during the next 15 months.

b) *Spouses of MAGs*
This category includes spouses of the MAGs who have been interviewed in-depth.

The **FGDs** will be conducted separately with MAGs, their spouses and elderly women and influential community members.

Each KII, IDI, and FGD will take around one and a half hours. All interviews will be digitally recorded with consent of the respondents. We will collect three rounds of qualitative data throughout the study period. Table 1 describes the methods, participants, procedure, and tentative sample size.

Table 1: Qualitative methods and participants

Methods	Participants	Purpose	Baseline	Endline
Method 1: Key Informant Interview (KII)				
KII with mothers-in-law of MAGs, NGO staff, CHWs, village doctor/ traditional healers/ drug sellers.	2.1. MiLs of MAGs<br><br>2.2. NGO staff<br><br>2.3. Government CHWs (e.g., FWV) 2.5.<br><br>2.4. Village doctors/drug sellers/ traditional healers	This method will generate information on empowerment, FP, child bearing including first conception, and DV of MAGs and social norms around these.	6	6
Method 1: In-depth Interviews				
In-depth interviews with MAGs and their spouses	1.1. MAGs aged 15-19<br><br>1.2. Spouse of MAGs aged 15-19	This method will generate information on knowledge, attitudes and practices regarding SRHR of MAGs' with a focus on empowerment and consent and choice and social norms around these.	16 MAGs and<br><br>16 spouses of MAGs	No. of MAGs and spouses retained in the cohort
Method 3: Focus Group Discussion (FGD)				

FGD with MAGs, their spouses, and elderly women and influential community members aged 35-59 years.		This method will generate information on knowledge, attitudes and practices regarding SRHR of MAGs' with a focus on empowerment and consent and choice and social norms around these.	2 with MAGs;<br><br>2 with spouses of MAGs;<br><br>2 with elderly women;<br><br>2 with influential community members	2 with MAGs;<br><br>2 with spouses of MAGs;<br><br>2 with elderly women;<br><br>2 with influential community members
Total			46	46 (max)

Qualitative data collection tools, data collection and management

The qualitative data collectors will have Masters degree in anthropology or any social science discipline. They will receive an 8-day training on gender, rights, empowerment, SRHR, communication, negotiation, decision-making, social norms, qualitative research methods, and research ethics. Interviewers will be gender matched with the informants. The end line will follow the same strategy enabling us to assess changes over time in the cohort of group members and in the community over time.

Sample Size Calculation and Outcome (Primary and Secondary) Variable(s)

Clearly mention your assumptions. List the power and precision desired. Describe the optimal conditions to attain the sample size. Justify the sample size that is deemed sufficient to achieve the specific aims.

The sample size for the survey of MAGs was calculated based on the primary outcomes "ability of a MAG to achieve own choice regarding: (1) FP use; (2) conception; and (3) SRH service uptake". Considering a cluster size of 22 married adolescent girls and their husband, and assuming a 50% prevalence rate of ability of a MAG to achieve own choice in SRH issues among this group (as unknown), 15% effect size, an Intra-Cluster Correlation of 0.05, 5% significance level and 80% power we required 16 clusters per arm making the total number of clusters 32. Considering a 10% non-response rate at baseline, 20% refusal rate among MAGs interviewed at baseline to participate in the intervention, and another 15% drop out rate at endline among the MAGs participated in the intervention, the group size increased to 35 and total sample size increased to 1,120 MAGs. As these considerations provided us with the maximum sample size, this current sample size will allow us to measure the changes in all other secondary outcomes of the study.

The husband of the selected MAGs will also be included in the study to measure the secondary outcomes related to their knowledge and attitudes regarding SRHR relevant to them and the MAGs.

To assess social norm change we focused on social norms around the MAGs' consent and choice in SRH. We will assess this change surveying adult community members aged 35-59 years. Since the prevalence of positive social norms around consent and choice in SRH issues is unknown among this population we assumed it as 50%. Considering a 15% effect size, 5% significance level, 80% power and 5% non-response rate, we required 540 community members (270 females and 270 males) from 32 clusters. Dividing the numbers by number of clusters and then rounding up the total sample size increased to 576 (nine females and nine males from each cluster).

The qualitative sample size includes 6 KIIs, 32 IDIs and 8 FGDs at baseline and endline.

Study outcomes and measurement of study variables

The study outcomes will be measured after the completion of the intervention implementation. The primary outcome of this study is - increase in instrumental agency of MAGs in decision making regarding SRHR (i.e., FP use; timing of first conception; and number of children to have). Table 2 presents the primary and secondary outcomes to be measured among different target groups.

Table 2. Different outcomes to be measured among different target groups of Balika Bodhu

Sl	Outcome	MAGs	Husbands of MAGs	Community members
**Primary outcome**				
1	Instrumental agency of MAGs in decision making regarding SRHR (i.e., FP use; timing of first conception; and number of children to have)	√		
**Secondary outcomes**				
2	Intrinsic agency of MAGs	√		
3	Instrumental agency of MAGs	√		
4	Collective agency of MAGs	√		
5	Critical consciousness (perceived inequality, egalitarianism and critical action) regarding gender inequality in the society	√		
6	Knowledge of MAGs regarding FP and conception	√		
7	Positive attitudes of MAGs regarding FP and conception	√		
8	Couple communication initiated by MAGs regarding FP use, timing of first conception, and number of children to have	√		
9	MAGs attempts to negotiate FP use, timing of first conception, and number of children to have as per her choice	√		
10	Service uptake among MAGs regarding abortion and post-abortion care, and help seeking after experiencing violence	√		
11	Prevalence of intimate partner violence (IPV) among the MAGs	√		
12	Knowledge of husbands of MAGs regarding FP and conception		√	
13	Positive attitudes of husbands of MAGs regarding FP and conception		√	
14	Positive attitudes regarding agency of MAGs regarding SRHR among the husbands		√	
15	Non-condoning attitudes of husbands of MAGs regarding violence against women		√	
16	Positive attitudes of husbands of MAGs regarding gender roles within household		√	
17	Knowledge of community members regarding FP and conception			√
18	Positive attitudes of community members regarding FP and conception			√
19	Positive attitudes regarding agency of MAGs regarding SRHR among the community members			√
20	Positive attitudes of community members regarding violence against women			√
21	Positive attitudes of community members regarding gender roles within household			√
22	Positive social norms regarding SRHR of MAGs	√		√

Instrumental agency of MAGs in decision making regarding SRHR (i.e., FP use; timing of first conception; and number of children to have)

The MAGs will be asked about the main decision maker regarding: (a) FP use; (b) timing of first conception; and (c) number of children to have. The response options included '1=the MAG herself', '2=husband', '3=jointly' and '4=someone else'. A follow up question, 'what kind of joint decision it was', was asked if the respose was 'jointly'. The response options included '1=he voiced his choice and I agreed', 'he voiced choice and forced me to agree', and '3=I voiced my choice and he agreed'. A MAG will be considered agentic in decision making regarding a specific theme of SRHR and will be coded as 'Yes=1' if she decided herself or it was a joint decision without any force on her, and 'No=2' otherwise. We will generate a summative score, with a higher score indicating higher agency.

Women's empowerment

We will measure empowerment of MAGs through measuring agency using The Women's Agency Scale 61 (WAS-61) (Yount et al., 2020).

Intrinsic agency

We will assess women's intrinsic agency, using a validated, 29-item three-dimensional measure capturing women's intrinsic voice and mobility; gender equitable attitudes; and non-justification of wife beating (CFA standardized factor loadings: 0.54–0.81, 0.35–0.82, and 0.87–0.93, respectively; CFI = 0.94; TLI = 0.93; RMSEA = 0.04) (Yount et al., 2020.

Intrinsic voice and mobility will be measured using nine items capturing women's level of comfort (1 = not at all to 3 = very) going places alone, like the 'home of a relative', and expressing opinions with community members, like government officials, NGO officials, and community leaders. A summative score will be obtained, with a higher score indicating higher intrinsic voice and mobility.

Gender equitable attitudes will be measured using 14 items capturing women's agreement (1 = strongly disagree to 4 = strongly agree) with items, like "a woman should obey her husband," and "a couple should decide together if they want to have children." Negatively valenced items will be reverse-coded, so higher score indicates stronger endorsement of more equitable attitudes.

Non-justification of wife beating will be measured using six items capturing responses (1 = strongly disagree to 4 = strongly agree) to the question, 'Does a man have a good reason to hit his wife' if, for example, she 'disobeys him' or "rudely argues with him'. Items will be reverse-coded, so higher scores indicates stronger non-justification of wife beating.

Instrumental agency

We will assess women's instrumental agency using a validated, 17-item three-dimensional measure capturing women's use of financial services, voice with husband, and voice and mobility outside the home (CFA standardized loadings 0.57–0.95, 0.48–0.99, and 0.43–0.79, respectively, CFI = 0.94, TLI = 0.93, RMSEA = 0.05).

Five items will be used to measure women's frequency of using (1 = never to 4 = often) financial services, such as 'money deposit' and 'checking account'. A summative score will be obtained, with a higher score indicating more frequent use of financial services.

Three items will capture women's level of influence (1 = none to 4 = total) with their husband about 'how to spend money your husband gives you' and 'how to spend your husband's earnings' and their frequency of expressing opinions (1 = never to 4 = often) with husbands. A summative score will be obtained, with a higher score indicating higher level of influence with husband.

Nine items will be used to measure women's frequency (1 = never to 4 = often) of going places like 'the home of a relative' and expressing opinions with community members like government officials and community leaders. A summative score will be obtained, with a higher score indicating more frequent mobility and expressing opinions with community members.

Collective agency

We will assess women's collective agency regarding influence in the community using an eight-item validated scale (CFA standardized loadings 0.52–0.84; CFI = 0.96; TLI = 0.95; RMSEA = 0.10) and regarding women's leadership in groups using a validated seven-item scale (CFA standardized loadings: 0.54–0.83; CFI = 0.96; TLI = 0.95; RMSEA = 0.10). The participants will report their agreement (1 = totally disagree to 4 = totally agree) with statements like 'women like me can really understand what is going on with my community' and 'I am often a leader in groups', 'I prefer to be a leader rather than a follower', and 'Other people usually follow my ideas', etc. (Yount et al., 2020; Hinson et al., 2016). Summative scores will be obtained, with higher scores indicating stronger collective agency.

Critical Consciousness of MAGs related to their consent and choice in SRH

Critical Consciousness (CC) is theorized to be composed of two subcomponents. One of them is critical reflection, which will encompass the ability to critically reflect on perceived societal inequalities as well as the endorsement of societal equality. The second component is critical action, which encompasses individual and/or collective action taken to change perceived social inequalities (Freire, 1993). We will measuree critical consciousness of MGAs using the validated 15-item Critical Consciousness Scale (CCS) (Mandal et al., 2020). Ten critical reflection related items will be used to measure the perceived inequality and egalitarianism. A typical example include 'Women have fewer opportunities than men'. The response options include: strongly agree, agree, disagree and strongly disagree. Another five items will be used to assess critical action, or the degree to which they have participated in individual and/or collective action to produce sociopolitical change, with the response options – not at all, rarely, sometimes and often. A summative score will be obtained, with a higher score indicating higher critical consciousness.

Knowledge, attitudes, communication, negotiation and practices of MAGs regarding SRHR

The MAGs will be asked about their knowledge, attitudes, practices, needs, consent, choice, communication and negotiation regarding FP use, conception and SRH service uptake. The questions will heavily draw upon the standard Demographic and Health Survey questionnaire (NIPORT & ICF, 2019), SAFE (Naved et al., 2018), IMAGINE and AdSEARCH FGWs Cohort questionnaires.

Knowledge of MAGs regarding FP and conception

The MAGs knowledge on number of modern methods, and emergency contraceptive pill will be measured using two questions: i) which of the FP methods have you heard of; and ii) have you heard about emergency contraceptive pill. A conitnuos variable will be created for the first question based on the number of correct answers, while a binary variable will be created from the resposes on the second question by recoding to '1=Yes' if the respondent heard of emergency contraceptive pill, and '0=No' otherwise.

The MAGs knowledge of regarding conception will be assessed using the following four questions: i) what is the appropriate age at first conception for a woman; ii) if a girl gets pregnant in her teenage years, what are the potential problems she might have to face; iii) after the birth of a child can a woman become pregnant before her menstrual period has returned: and iv) what are the consequences of not having proper spacing between two pregnancies. For questions i) and iii), separate binary variables will be created from the resposes by recoding to '1=Yes' if it was a correct answer, and '0=No' otherwise. For questions ii) and iv), separate conitnuos variables will be created based on the number of correct answers.

A summative score will then be obtained by adding all the knowledge related variables, with a higher score indicating greater knowledge.

Positive attitudes of MAGs regarding FP and conception

Positive attitudes of MAGs regarding SRHR will be measured using six items, like 'family planning is a women's business and a man should not worry' and 'a woman has right to choose when to first conceive'. The participants will report their agreement on a Likert scale (1 = strongly agree to 4 = strongly disagree). A summative score will be obtained, with a higher score indicating more positive attitude regarding SRHR.

Increase in couple communication initiated by MAGs regarding FP use, timing of first conception, and number of children to have

The MAGs will be asked about any communication/discussion with husband regarding FP use, first conception, and number of children they want to have. The response options include "1=Yes" and "2=No". They will also be asked about who initiated the conversation, with the response options "1=herself", "2=husband" and "3=don't remember". Separate variables will be created from these resposes by recoding

"herself" as "1"; "husband" as "0" and "don't remember as "missing value". A summative score will be obtained by adding the number of conversations she initiated on different SRHR issues, with a higher score indicating higher initiated commnunication.

MAGs attempts to negotiate her choice of FP use, timing of first conception, and number of children to have

The MAGs will be asked about what happened if there was any mismatch between her and her husband's choice regarding the above mentioned SRHR themes. We will assess if she attempted to negotiate in such case and also if her husband tried to convince her to accept his own choice. In both cases, an to negotiate will be coded as '1', and '0' otherwise. A summative score will be obtained by accumulating the number of SRHR themes where she attempted to negotiate with a higher score indicating higher attempts.

Abortion and post-abortion service uptake and help seeking by physically and/or sexually abused MAGs

If any MAG reports having an induced abortion/MR, she will be asked about venue of the service received, provider and the method of abortion. The MAGs with an abortion will be asked about post-abortion care. The MAGs who report experiencing any physical and/or sexual intimate partner violence will be asked about their help seeking. The results will be presented in frequencies and percentages.

Intimate partner violence (IPV)

MAGs' experience of IPV (i.e. controlling behaviour, and physical and sexual violence IPV) will be measured using a modified version of The Revised Conflict Tactic Scale (Straus et al., 1996). The scale is the most widely used measure of IPV which is characterized by having direct and behaviorally explicit questions in order to reduce variation in the interpretation and understanding of what violence comprises of. These instruments are designed to minimize reporting biases that arise from subjective perceptions of abuse by asking only about specific behaviours perpetrated by a male partner (e.g., "Has your husband or any other family member ever slapped you or threw something at you that could hurt you?). Questions will capture behaviours that reflect minor and severe physical violence (e.g., slapped, pushed, hit with a fist, kicked, dragged, choked, burned, threatened with a weapon); and sexual violence (e.g., forced sexual intercourse or other sexual acts). To measure economic coercion, we will use a modified version of the ECS-20 (Yount et al., 2022). Typical items include—your husband or any other family member ever and in last 12 months: disallowed you to go to your work, school or training, or do any home-based income earning activity; told that you could work outside the home only if you kept up with the housework; told that you could earn income only if you worked from home; etc. A woman will be considered exposed to any specific type of IPV if she responds "yes" to any of the items related to that specific type of IPV, and will be coded "yes=1", and "no=0" otherwise.

Knowledge of husbands of MAGs regarding SRHR of MAGs

The knowledge of husbands of MAGs on number of modern methods, and emergency contraceptive pill will be measured using two questions: i) which of the FP methods have you heard of; and ii) have you heard about emergency contraceptive pill. A conitnuos variable will be created for the first question based on the number of correct answers, while a binary variable will be created from the resposes on the second question by recoding to '1=Yes' if the respondent heard of emergency contraceptive pill, and '0=No' otherwise.

The knowledge of husbands of MAGs regarding conception will be assessed using the following four questions: i) what is the appropriate age at first conception for a woman; ii) if a girl gets pregnant in her teenage years, what are the potential problems she might have to face; iii) after the birth of a child can a woman become pregnant before her menstrual period has returned: and iv) what are the consequences of not having proper spacing between two pregnancies. For questions i) and iii), separate binary variables will be created from the resposes by recoding to '1=Yes' if it was a correct answer, and '0=No' otherwise. For questions ii) and iv), separate conitnuos variables will be created based on the number of correct answers.

A summative score will then be obtained by adding all the knowledge related variables, with a higher score indicating greater knowledge.

Positive attitudes of husbands of MAGs regarding SRHR

Attitudes of husbands of MAGs regarding SRHR will be measured using six items, like "family planning is a women's business and a man should not worry" and "a woman has right to choose when to first conceive". The participants will report their agreement on a Likert scale (1 = strongly agree to 4 = strongly disagree). A summative score will be obtained, with a higher score indicating more positive attitude regarding SRHR.

Positive attitudes regarding agency of MAGs regarding SRHR among their husbands
Attitudes of husbands of MAGs about agency of MAGs regarding SRHR will be measured using 12 items. A typical example includes "It should be a wife's decision when to have a child". The participants will report their agreement on a 4-point Likert scale (1 = strongly agree to 4 = strongly disagree). A summative score will be obtained, divided into tertiles. Negatively valenced items will be reverse-coded, so higher score indicates more positive attitudes.

Positive attitudes of husbands of MAGs regarding violence against women
Attitudes of husbands of MAGs regarding violence against women will be measured using six items. A typical example includes "There are times when a woman deserves to be beaten". The participants will report their agreement on a 4-point Likert scale (1 = strongly agree to 4 = strongly disagree). A summative score will be obtained, with a higher score indicating more positive attitudes.

Positive attitudes of husbands of MAGs regarding gender roles within household
Attitudes of husbands of MAGs regarding gender roles within household will be measured using three items. A typical example includes "Child care activies such as cleaning after toilet, giving bath is a mother's responsibility". The participants will report their agreement on a 4-point Likert scale (1 = strongly agree to 4 = strongly disagree). A summative score will be obtained, with a higher score indicating more positive attitudes.

Knowledge of community member regarding SRHR of MAGs
The community members' knowledge on number of modern methods, and emergency contraceptive pill will be measured using two questions: i) which of the FP methods have you heard of; and ii) have you heard about emergency contraceptive pill. A conitnuos variable will be created for the first question based on the number of correct answers, while a binary variable will be created from the resposes on the second question by recoding to '1=Yes' if the respondent heard of emergency contraceptive pill, and '0=No' otherwise.

The community members' knowledge regarding conception will be assessed using the following four questions: i) what is the appropriate age at first conception for a woman; ii) if a girl gets pregnant in her teenage years, what are the potential problems she might have to face; iii) after the birth of a child can a woman become pregnant before her menstrual period has returned: and iv) what are the consequences of not having proper spacing between two pregnancies. For questions i) and iii), separate binary variables will be created from the resposes by recoding to '1=Yes' if it was a correct answer, and '0=No' otherwise. For questions ii) and iv), separate conitnuos variables will be created based on the number of correct answers.

A summative score will then be obtained by adding all the knowledge related variables, with a higher score indicating greater knowledge.

Positive attitudes of community members regarding SRHR
Attitudes of community members regarding SRHR (FP and conception) will be measured using six items, like "family planning is a women's business and a man should not worry" and "a woman has right to choose when to first conceive". The participants will report their agreement on a Likert scale (1 = strongly agree to 4 = strongly disagree). A summative score will be obtained, with higher score indicating more positive attitude regarding SRHR.

Positive attitudes regarding agency of MAGs regarding SRHR among their husbands
Attitudes of community members about agency of MAGs regarding SRHR will be measured using 12 items. A typical example includes "It should be a wife's decision when to have a child". The participants will report their agreement on a 4-point Likert scale (1 = strongly agree to 4 = strongly disagree). A summative score will be obtained, divided into tertiles. Negatively valenced items will be reverse-coded, so higher score indicating more positive attitudes.

Positive attitudes of husbands of MAGs regarding violence against women

Attitudes of community members regarding violence against women will be measured using six items. A typical example includes "There are times when a woman deserves to be beaten". The participants will report their agreement on a 4-point Likert scale (1 = strongly agree to 4 = strongly disagree). A summative score will be obtained, with higher score indicating more positive attitudes.

Positive attitudes of husbands of MAGs regarding gender roles within household

Attitudes of community members regarding gender roles within household will be measured using three items. A typical example includes "Child care activies such as cleaning after toilet, giving bath is a mother's responsibility". The participants will report their agreement on a 4-point Likert scale (1 = strongly agree to 4 = strongly disagree). A summative score will be obtained, with higher score indicating more positive attitudes.

Community social norms related to consent and choice of MAGs regarding their SRH

We will measure Social norms around consent and choice of MAGs in SRH following Cialdini (1998) and Bicchieri (2006, 2012) [32-34]. The statements to capture social norms around consent and choice of MAGs regarding their SRH will be framed considering Cialdini's (1998) and Bicchieri's (2006, 2012) theory of social norms and validated for the target population. The theory of social norms is a theory of what motivates collective patterns of behavior. It tries to answer a very basic question – why do people do what they do? The key concepts in this theory include empirical and normative expectations.

Empirical expectations refer to "What most people in her/his village will do in a specific circumstance." A typical example includes: "Do you strongly agree/agree/disagree/strongly disagree with this statement - Most of the married adolescent girls in your village achieve their aspiration regarding their desired method of contraception".

Normative expectations refer to "What most people in her/his village expect others to do in a specific circumstance." A typical example includes: "Do you strongly agree/agree/disagree/strongly disagree with this statement - Most of the husbands of married adolescent girls in your village will approve if their wives try to achieve their aspiration regarding their desired method of contraception".

Social norms around SRHR of MAGs in the domains such as FP, conception, number of children to have, abortion and SRH service uptake will be assessed using data from the MAG and community surveys. A set of statements will be used to assess the participants' level of agreement collected on a Likert scale (1 = totally disagree to 4 = totally agree). A scale will be constructed and validated using factor analysis. A summative score will be obtained, with a higher score indicating stronger positive social norm.

Data Analysis

Describe plans for data analysis, including stratification by sex, gender and diversity. Indicate whether data will be analysed by the investigators themselves or by other professionals. Specify what statistical software packages will be used and if the study is blinded, when the code will be opened. For clinical trials, indicate if interim data analysis will be required to determine further course of the study.

Quantitative data analyses

Items in the survey questionnaires will be recoded so that all are anchored at 0. For example, for gender norms and attitudes, each item with scores ranging between 1 and 4 in the questionnaire will be recoded to 0–3. Negatively valenced items will be reverse-coded, so higher score indicates positive outcome. All the measurement scales used in the study will be validated using factor analysis. This exercise will allow us as well to reduce the number of items in the scales. Internal consistency (or reliability) of a scale will be measured using Cronbach's alpha. A scale with alpha equal to 0.60 or more will be considered acceptable. Scale validity will be measured using the Kaiser-Meyer-Olkin (KMO) test, where KMO equal to 0.60 or more will be considered acceptable. Once the final scale is constructed, a summative score will be obtained for each scale, and the scores will then be divided into tertiles.

During baseline we will explore the current situation in empowerment and SRHR among MAGs and their correlates, while at end line we will measure the impact of the social norms intervention on empowerment, on social norm change and promotion of demand, access and positive practices in relation to SRHR among the MAGs. Using the baseline survey data, the basic background characteristics (e.g., age, education,

religion, ethnic group) of the girl and community samples will be compared by arm (control, treatment) to check the arm balance. Descriptive analyses will be performed to report frequencies and percentages of different outcomes of interest. Chi-square, ANOVA and t-tests will be performed to check whether the study arms are covariate balanced. Linear and logistic regression analyses will be performed to identify the underlying factors for current situation in SRHR among MAGs.

Intention-to-treat (ITT) analysis will be used during assessing the impact of the intervention. Thus, all the MAGs enrolled at baseline and could be successfully interviewed at endline will be included in the analysis irrespective of their actual level of participation in the intervention activities. The information on participation of study participants in the intervention activities will come from the programme monitoring data and will also used in the models. The impact of the intervention on primary and secondary outcomes will be assed using risk ratios/regression coefficients derived from binary/linear regression analyses for measuring change in outcomes in the intervention arms relative to change in the control arm. Significance level will be set at $p<.05$ for all analyses. The data will be analysed using STATA 16.

Qualitative data analyses
With the informants' permission, all interviews and FGDs will be digitally recorded. All recorded interviews will be transcribed verbatim. Data analysis will be iterative. To explore research questions, we will combine the intense, within-case focus of Narrative Analysis with the across-case approach of Grounded Theory, a robust analytic strategy for qualitative data.

Narrative analysis will be used with the IDIs to examine each transcript for a core narrative or 'story' of each MAGs and their husbands about how they experience and view gender; restrictions and barriers in fulfilling their aspirations and expectations; privileges and discrimination; agency and/or the lack of it; social norms and practices around SRHR of MAGs (e.g., timing of first conception, use of FP methods to delay first conception and for birth spacing, unwanted pregnancy, etc. This approach maintains the issues discussed in the life context of an individual. We then will compare core narratives across participants to identify distinct and shared features of the narratives. Narrative analysis will identify, for example, the baseline status of the MAGs and their husbands in the mentioned areas. A comparison of the IDIs of the same MAGs and their husbands at baseline and end line will provide us with insights into change that relates to Balika Bodhu.

Grounded Theory *(GT)* is well suited 1) to identify cross-cutting themes and normative constructs related to our areas of investigation and 2) to link these themes into an explanatory framework to understand more fully the context of social norms and practices around SRHR of MAGs (Corbin & Strauss, 2008; Charmaz, 2006). We will follow recommended steps for GT analysis: 1) *Identify themes and develop a codebook.* We will systematically read, memo, and discuss themes raised in the IDIs, KIIs and FGDs. Core themes will be developed into a codebook, listing each theme and how to label data for each theme. The codebook will include inductive themes emerging from the data and deductive themes developed a priori from theoretical domains in the interview guides. 2) *Code data*. Using themes from the codebook, we will code the full dataset using textual data analytic software that permits cross-classification and retrieval of transcripts and segments of text by theme. Key personnel will review the coding regularly to monitor quality. 3) *Conduct descriptive and comparative analysis.* Descriptive analysis will be conducted to identify the types, contexts, and nuances in the areas of investigation. We will try to reveal patterns in the data, with issues mentioned repeatedly across interviews, and by certain sub-groups, suggesting normative perceptions or behaviour. 4) *Develop conceptual framework of findings.* Findings from the descriptive and comparative analysis will be developed into a conceptual framework that explains local constructs, the context and social norms which influence the decision making, consent and choice around SRHR of MAGs We will use supporting evidence and counter-evidence (Strauss, 1987). To test our conceptual model, which we will use to contextualize and interpret survey findings. 5) We will *triangulate data from all qualitative and quantitative data sources*.

Data Safety Monitoring Plan (DSMP)

All clinical investigations (research protocols testing biomedical and/or behavioural intervention(s)) should include the Data and Safety Monitoring Plan (DSMP). The purpose of DSMP is to provide a framework for appropriate oversight and monitoring of the conduct of clinical trials to ensure the safety of participants and the validity and integrity of the data. It involves involvement of all investigators in periodic assessments of data quality and timeliness, participant recruitment, accrual and retention, participant risk versus benefit, performance of trial sites, and other factors that can affect study outcome.

N/A

Ethical Assurance for Protection of Human rights

Describe the justifications for conducting this research in human participants. If the study needs observations on sick individuals, provide sufficient reasons for using them. Indicate how participants' rights will be protected, and if there would be benefit or risk to each participants of the study. Discuss the ethical issues related to biomedical and social research for employing special procedures, such as invasive procedures in sick children, use of isotopes or any other hazardous materials, or social questionnaires relating to individual privacy. Discuss procedures safeguarding participants from injuries resulting from study procedures and/or interventions, whether physical, financial or social in nature. [Please see Guidelines]

Ethical considerations

The study will seek ethical approval from icddr,b's institutional review board. Informed consent will be obtained from the study participants. Participation in the study will be as entirely voluntary. Individual verbal consent will be sought by the interviewer prior to the interview with each study participant. The MAGs who are minor (aged below 18) will be considered as "mature minors" and following the guidelines for adolescent health research published by the National Commission for the Protection of Human Subjects of Biomedical and Behavioral Research and the Society for Adolescent Medicine (Society for Adolescent Medicine, 2003; National Commission for the Protection of Human Subjects of Biomedical and Behavioral Research, 1977) we will seek informed consent from them.

According to English et al., (1995), mature minors are those who are living apart from their parents, married, pregnant, parents themselves, and self-supporting and they are allowed to give consent (English et a., 1995). In our study, we will consider MAGs aged below 18 as "mature minors" as all of them are married and some of them are expected to be pregnant and/or have child/ren. Thus, the mature minors in this study do not require consent from a guardian.

The primary ethical purpose of obtaining consent of a guardian is to ensure that vulnerable children are protected from potential risks a research may pose. However, this approach does not always protect a minor (National Commission for the Protection of Human Subjects of Biomedical and Behavioral Research, 1977), and may be problematic in some situations as well (Department of Health and Human Services, National Institutes of Health, Office for Protection from Research Risks, 2001). Examples of such situations, where parental permission is not appropriate and is not a reasonable requirement include, abuse, contraception use, etc.). Most of the MAGs will be living not with their parents, but with their husbands and in-laws, usually considered as their guardians. Since our study deals with sensitive issues such as sexual and reproductive coercion and violence, where the most common perpetrators are the husbands it would be unethical to leave it up to them to decide whether or not the girl will participate in the study. Informing the potential perpetrator about the study may also increase the risks of abuse of the girls. Due to these ethical concerns consent must not be obtained from the husbands.

All the participants will be informed orally of the purpose and nature of the study, its expected benefits, sensitivity, confidentiality and voluntary nature of participation.

This study will be guided by CIOMS International Guidelines for Ethical Review of Epidemiological Studies (CIOMS and WHO, 2002) and the WHO recommendations for ethical considerations in researching violence against women (WHO, 2001). This application and all documents related to the protection of human subjects will be reviewed the IRB of icddr,b. All members of the research team will be trained to meet the highest ethical standards of data collection and analysis. Specifics of this have been presented below.

Informed consent

The selected participants will be contacted in-person using information collected through household enumeration. The participant will be interviewed in private and will be informed orally of the purpose and nature of the study, its expected risks and benefits, and voluntary nature of participation. As part of the consent procedure, the participant will be informed that the data collected will be held in strict confidence. To ensure that the participant is aware that the survey includes questions on highly personal and sensitive topics, the interviewer will forewarn the participant that some of the topics are difficult to talk about. The respondent will be free to terminate the interview at any point, and to skip any questions that she does not

wish to respond to. The interviewer will then record on the consent form that the consent procedure has been administered, and note whether permission to conduct the interview has been granted.

Voluntary participation
Participation in the study will be on a voluntary basis. No inducements will be made. The participant will be told that s/he is free not to participate in the study; to terminate the interview at any point, and to skip any questions that s/he does not wish to respond to.

Confidentiality
A number of mechanisms will be used to protect the confidentiality of the information collected:

All interviewers will receive strict instructions about the importance of maintaining confidentiality. No interviewer will conduct an interview in their own community. No names and addresses will be recorded on the tablets. Instead, all the study participants will be given a unique code and all the identifying information will be kept in a separate file for follow up visits. The file will exclusively be accessed by the researchers and will only be used to contact the respondent for research purpose. The researchers will use pseudonyms and unique numbers for identifying the informants. All the data and digital recordings that are made during the qualitative interviews will be kept on a password protected computer with limited access only researchers involved with this project. De-identified data will be provided to the transcribers. Recordings will be destroyed upon completion of the study (we will keep them available during the data analysis in case questions arise that require a review of the recording).

For both the quantitative and qualitative study, the de-identified data will be analysed and care will be taken to present the research findings in sufficiently aggregated form to ensure that no study participant can be identified.

Risks and Benefits
The study participants will be informed of expected risks and benefits of the study. For example, the time and effort that s/he needs to devote to participate in the interview, s/he may find it difficult to answer one or more questions, benefits of her information to help research and policy, etc.

Time required and principle of compensation
The study participants will be clearly informed about the time required to complete the interview, and that they will not receive any compensation in terms of money or any other thing for participating in this survey.

Security and safety of study participants
If the sensitive issues explored in the research becomes widely known - either within the household or among the wider community – both the MAGs giving interviews and the study team may be at risk of harm. Thus, the research will be introduced at the local and household levels as a study of young women's reproductive health and life experiences.

To assure safety of the married MAGs the spouses of the MAGs will not be asked about DV. Care will be taken to phrase the questions in the qualitative guides for the spouses in a manner to avoid sensitivities. All interviews will only be conducted in private in a location of the participant's choice. If confidentiality cannot be maintained for any reason the interview will be rescheduled (or relocate) at a time (or place) that may be more safe or convenient for the respondent.

Interviewers will be trained to terminate or quickly change the subject of discussion if an interview is interrupted by anyone. During the interview, the interviewer will forewarn the respondent that she will terminate or change the topic of conversation if the interview is interrupted, and will be able to skip to these questions at any point if needed. To ensure that interviewers gain experience about how to handle interrupted interviews, their training will include a number of roles play exercises simulating different situations that they may encounter.

Do no harm and respect individual's decisions and choices
Interviewers will be trained to be aware of any adverse effect of data collection, and, if necessary, will terminate the interview if the effect seems too negative. Care will be taken when designing the questionnaire to try to carefully and sensitively introduce and enquire about different sections of the questionnaire, starting

from the less sensitive one to more sensitive sections. Each interview will aim to end in a positive manner, which provides the participant with a positive outlook and reinforces his/her coping strategies. The questionnaire will include scripted conclusion for the interview stressing the importance of the information that s/he has provided, making comment on the respondent's strengths, and highlighting the unacceptability of her adverse experiences. Extensive training will be provided to the survey team. The training will not only discuss survey techniques, but also ethical issues, how to respond to, and if necessary, provide support to the participants. The interviewers will collect information on available help seeking services on SRHR, and DV while they will be working in each cluster. All the participants will be provided with the necessary information for help seeking regarding SRHR, and DV.

Mechanisms to attend to researchers' and field workers' needs

A number of mechanisms will be adopted to attend to the needs of researchers and field workers. During the training process these issues will be openly presented, and participants will be given the option of withdrawing from the project without prejudice. During the research regular debriefing meetings will be scheduled to enable the research team to discuss what they are hearing, their feelings about the situation, and how it is affecting them. These meetings will aim to reduce the stress of the field work, and avert any negative consequences. This strategy proved effective in the Bangladesh component of the WHO multi-country study conducted by icddr,b.

Despite these measures, some field workers may need to be given less emotionally taxing tasks, be given a break from the study or to withdraw from the research altogether. To account for these possibilities, sufficient numbers of field workers will be recruited to allow for a 10% attrition rate of interviewers over the study.

Harmful publicity

The study findings will be disseminated in a scientifically rigorous manner. Particular attention will be paid to ensuring that the findings are not used as a means to describe one setting or group as being "worse" than another.

Use of Animals

Describe if and the type and species of animals to be used in the study. Justify with reasons the use of particular animal species in the research and the compliance of the animal ethical guidelines for conducting the proposed procedures.

N/A

Collaborative Arrangements

Describe if this study involves any scientific, administrative, fiscal, or programmatic arrangements with other national or international organizations or individuals. Indicate the nature and extent of collaboration and include a letter of agreement between the applicant or his/her organization and the collaborating organization.

NA

Facilities Available

Describe the availability of physical facilities at site of conduction of the study. If applicable, describe the use of Biosafety Level 2 and/or 3 laboratory facilities. For clinical and laboratory-based studies, indicate the provision of hospital and other types of adequate patient care and laboratory support services. Identify the laboratory facilities and major equipment that will be required for the study. For field studies, describe the field area including its size, population, and means of communications plus field management plans specifying gender considerations for community and for research team members.

NA

Literature Cited

Identify all cited references to published literature in the text by number in parentheses. List all cited references sequentially as they appear in the text. For unpublished references, provide complete information in the text and do not include them in the list of Literature Cited. There is no page limit for this section, however, exercise judgment in assessing the "standard" length.

Addis Continental Institute of Public Health. Improving adolescent reproductive health and nutrition through structural solutions in West Hararge, Ethiopia (Abdiboru Project). 2017. https://www.careevaluations.org/wp-content/uploads/Abdiboru-Baseline-Quantitative-report-V5_03092017_Final-2.pdf

Addis Continental Institute of Public Health. Assessment of pathways and reasons for TESFA project Sustainability and Scalability. (TESFA+ Investigative Research. 2018. South Gondar, Amhara Region, Ethiopia

Agarwala R, Lynch SM. Refining the measurement of women's autonomy: an international application of a multi-dimensional construct. Social Forces. 2006. 84(4):2077-98.

Bandura A. Exercise of human agency through collective efficacy. Current directions in psychological science. 2000;9(3):75-8.

Bangladesh Bureau of Statistics (BBS) and UNICEF, 2019. Progotir Pathey, Bangladesh Multiple Indicator Cluster Survey 2019, District Summary Findings, Dhaka, Bangladesh: Bangladesh Bureau of Statistics (BBS).

Barua A, Kurz K. Reproductive health-seeking by married adolescent girls in Maharashtra, India. Reproductive Health Matters. 2001;9(17):53-62.

Bell DC and Cox ML. Social Norms: Do We Love Norms Too Much? Journal of Family Theory Review 2015 3 01;7(1):28–46. PubMed: 25937833

BLAST. Promoting Rights through Inclusion and Empowerment. 2022. https://www.blast.org.bd/index.php?option=com_content&view=article&id=623

Bicchieri C. The grammar of society: The nature and dynamics of social norms. 2006. New York: Cambridge University Press. 91.

Bicchieri C. Norms, conventions, and the power of expectations. In N. Cartwright & E. Montuschi (Eds.), *Philosophy of Social Science*. 2012. New York: Oxford University Press.

SDG Tracker: Bangladesh's Development Mirror. 2020. https://sdg.gov.bd/page/indicator-wise/1/43/2/0#1

Campbell C, MacPhail C. Peer education, gender and the development of critical consciousness: participatory HIV prevention by South African youth. *Social Science & Medicine.* 2002. 55:331–345.

CARE. IMAGINE Baseline Analysis Report. FAR HARBOR, Analysis Research. 2019

CARE. Inspiring Married Adolescent Girls to Imagine New Empowered Futures (IMAGINE). 2022. https://www.care.org/our-work/health/adolescent-health/imagine/)

Cepal N. Montevideo consensus on population and development. 2013. http://repositorio.cepal.org/handle/11362/40336 .

Cialdini RB, & Trost MR. Social Influence: Social Norms, Conformity and Compliance. In DT Gilbert, ST Fiske, & G Lindzey (Eds.), *The Handbook of Social Psychology*, fourth edition.1998. pp. 151-192. 1998.

CIOMS and WHO. International ethical guidelines for biomedical research involving human subjects. Geneva: CIOMS. 2002.

Cislaghi B, Denny EK, Cissé M, Gueye P, Shrestha B, Shrestha PN, Ferguson G, Hughes C, Clark CJ. Changing Social Norms: the Importance of "Organized Diffusion" for Scaling Up Community Health Promotion and Women Empowerment Interventions. Prevention Science. 2019. 20:936–946. https://doi.org/10.1007/s11121-019-00998-3

Cislaghi B and Heise L. Four avenues of normative influence. Health Psychology 2018.

Cisse M, Gueye P, Manel V. A community-led approach to community empowerment in Mali, Mauritania, Guinea, and Guinea-Bissau. Endline evaluation brief. 2018. Dakar: Tostan.

Clark CJ., Spencer RA, Shrestha B, Ferguson G, Oakes JM, Gupta J. Evaluating a multicomponent social behaviour change communication strategy to reduce intimate partner violence among married couples: study protocol for a cluster randomized trial in Nepal. BMC Public Health. 2017. 17(1):75. https://doi.org/10.1186/s12889-016-3909-9

Coast A, Jones N, Francoise UM, Yadete W, Isimbi R, Gezahegne K, Lunin L. Adolescent Sexual and Reproductive Health in Ethiopia and Rwanda: A Qualitative Exploration of the Role of Social Norms. SAGE Open. 2019; 9(1). https://doi.org/10.1177/2158244019833587

Corcoran KE, Pettinicchio D, Young JTN. Perceptions of structural injustice and efficacy: Participation in low/moderate/high-cost forms of collective action. *Sociological Inquiry.* 2015. 85(3):429–461. doi: 10.1111/soin.12082.

Deb S, Kabir A, Kawsar L. Women's Empowerment and Regional Variation of Contraceptive Norms in Bangladesh. Int Q Community Heal Educ. 2011;31: 401–410

Denny, E., & Hughes, C. (2017). Measuring changes in social norms: Learning from voices for change. Voices for Change. Available at: http://www.v4c-nigeria.com/wp-content/uploads/2014/09/1624-V4C-LPMeasuring-Change-WEB.pdf. Accesses 6 Feb 2019.

Department of Health and Human Services, National Institutes of Health, Office for Protection from Research Risks. Code of Federal Regulations: Title 45-Public Welfare; Part 46: Protection of Human Subjects. November 13, 2001.

Eger C, Miller G, Scarles C. Gender and capacity building: A multi-layered study of empowerment. World Development. 2018;106:207-19.

English A, Matthews M, Palamountain C, et al. State Minor Consent Statutes: A summary. San Francisco: National Center for Youth Law, 1995.

Fukuda-Parr S. The human development paradigm: operationalizing Sen's ideas on capabilities. Feminist economics. 2003;9(2-3):301-17.

Freire P. Education for critical consciousness. New York: Seabury Press; 1973.

Freire, P. (1993). Pedagogy of the oppressed. New York, NY: Continuum.

Gayen K, Raeside R. Social networks and contraception practice of women in rural Bangladesh. Soc Sci Med. 2010;71: 1584–1592. pmid:20869146

Godha D, Hotchkiss DR, Gage AJ. Association Between Child Marriage and Reproductive Health Outcomes and Service Utilization: A Multi-Country Study From South Asia. Journal of Adolescent Health. 2013;52:552e558m. http://dx.doi.org/10.1016/j.jadohealth.2013.01.021

González L, Molestina M, Torres I, Melo LA. UNICEF. Application of a human rights based approach and gender mainstreaming strategy for work with adolescents in Latin America and the Caribbean. UNICEF Regional Office; 2012.

Grant, C. and Laterra, A., 2019. Impact Evaluation of CARE's Inspiring Married Adolescent Girls to Imagine New Empowered Futures (IMAGINE) Project. Registry for International Development for Impact Evaluations (RIDIE). Available at: 10.23846/ridie176

Haque SE, Rahman M, Mostofa MG, Zahan MS. Reproductive Health Care Utilization among Young Mothers in Bangladesh: Does Autonomy Matter? Women's Heal Issues. 2012;22.

Holly B. Shakya, John R. Weeks and Nicholas A. Christakis, SSM - Population Health, 2019; 9 https://doi.org/10.1016/j.ssmph.2019.100371

Igras S, Saldhana N, Becker-Benton A, Dagadu NA, Pirzadeh M. Scaling-up Norms-Focused Interventions for Adolescent and Youth Sexual and Reproductive Health: Current Practice and Reflections for Moving the Field Forward. Journal of Adolescent Health. 2019; 64:S10eS12

Islam MM, Mahmud M. Marriage Patterns and Some Issues Related to Adolescent Marriage in Bangladesh. Asia-Pacific Population Journal. 1996;11(3).

Jemal AD. Transformative consciousness: Conceptualization, scale development and testing (Unpublished doctoral dissertation). 2016. Rutgers University; NJ.

Jiménez Thomas, D., Harper, C. and George, R. Mobilising for change: how women's social movements are transforming gender norms. ALIGN Report. London: 2021. https://www.alignplatform.org/resources/reportmobilising

Kabeer N. Resources, agency, achievements: Reflections on the measurement of women's empowerment. Dev Change. 1999. 30(3):435-64.

Kabeer N. Gender equality and women's empowerment: A critical analysis of the third millennium development goal 1. Gender & Development. 2005. 13(1):13-24.

Kamal SM. Childbearing and the use of contraceptive methods among married adolescents in Bangladesh. Eur J Contracept Reprod Heal Care. 2012;17: 144–154.

Khader S. Beyond Autonomy Fetishism: Affiliation with Autonomy in Women's Empowerment. Journal of Human Development and Capabilities. 2016. 17(1):125-39.

Kishor S, Gupta K. Women's empowerment in India and its states: evidence from the NFHS. Economic and Political Weekly. 2004. 694-712.

Mackie G, Moneti F, Shakya H and Denny E. What are Social Norms? How are they measured? 2015.

Malhotra A, Schuler SR. Women's empowerment as a variable in international development. Measuring empowerment: Cross-disciplinary perspectives. 2005. 71-88.

MacQuarrie KLD. The Unfolding of Women's Empowerment over the Life Course in Madhya Pradesh, India: The Influence of Family Formation and Early Empowerment Resources. Paper for the XXVI IUSSP International Population Conference, September 2009, Marrakech, Morocco. 2009.

MacQuarrie KLD, Nahar Q, Khan R, Sultana M. Decision-making Contexts around Early Childbearing and Contraception among Young, Married Women in Bangladesh, Extended abstract for submission to Population Association of America annual meeting Washington, DC, March 31-April 2, 2015. 2016.

MacQuarrie KLD, Edmeades J. Whose Fertility Preferences Matter? Husbands' and Mothers'-in-law Influence on Women's Abortion Decision-making. Population Research and Policy Review. 2015;34:615–639. DOI 10.1007/s11113-015-9364-y

Mandal H, Treves-Kagan S, Mejia C. Validating measures of reproductive empowerment in Kenya. North Carolina: USAID and MEASURE Evaluation. 2020.

Mason KO. Measuring Women's Empowerment: Learning from Cross-National Research. In: Narayan D, editor. Measuring Empowerment: Cross-Disciplinary Perspectives. Washington, D.C.: World Bank; 2005. p. 89-102.

Miedema SS, Haardörfer R, Girard AW, Yount KM. Women's empowerment in East Africa: Development of a cross-country comparable measure. World Development. 2018;110:453-64.

Ministry of Health and Family Welfare, Bangladesh, Partnership for Maternal, Newborn & Child Health, WHO, World Bank and Alliance for Health Policy and Systems Research. Success Factors for Women's and Children's Health: Bangladesh. 2015.

Naripokkho. On universal access to sexual and reproductive rights: Bangladesh. Country profile. 2017. http://arrow.org.my/wp-content/uploads/2017/04/Bangladesh-Country-Profile-on-SRR.pdf

National Commission for the Protection of Human Subjects of Biomedical and Behavioral Research. Report and Recommendations: Research Involving Children. 1977. Washington, DC: US Government Printing Office, DHEW Publication No. (S) 77-0004.

National Institute of Population Research and Training (NIPORT), and ICF. Bangladesh Demographic and Health Survey 2017-18: Key Indicators. 2019. Dhaka, Bangladesh, and Rockville, Maryland, USA: NIPORT, and ICF.

Naved RT, Mamun MA, Mourin SA, Parvin K. A cluster randomized controlled trial to assess the impact of SAFE on spousal violence against women and girls in slums of Dhaka, Bangladesh. PLoS ONE. 2018; 13(6): e0198926. https://doi.org/10.1371/journal.pone.0198926

Naved RT, Mamun MA, Talukder A, Mahmud S, Parvin K, Kalra S, Laterra A. Impact of Tipping Point Initiative, a social norms intervention, in addressing child marriage and other adolescent health and behavioral outcomes in a northern district of Bangladesh. 2022. (Forthcoming)

Parvin K, Talukder A, Mamun MA, Kalra S, Laterra A, Naved RT, Tipping Point Initiative study team. A cluster randomized controlled trial for measuring the impact of a social norm intervention addressing child marriage in Pirgacha in Rangpur district of Bangladesh: study protocol for evaluation of the Tipping Point Initiative. Global Health Action. 2022 Dec 31;15(1):2057644.

Samandari, G., Sarker, B.K., Grant, C. et al. Understanding individual, family and community perspectives on delaying early birth among adolescent girls: findings from a formative evaluation in rural Bangladesh. BMC Women's Health 20, 169 (2020). https://doi.org/10.1186/s12905-020-01044-z

Santhya KG, Jejeebhoy SJ. Sexual and reproductive health and rights of adolescent girls: Evidence from low- and middle income countries. Global Public Health. 2015;10(2):189-221, DOI:10.1080/17441692.2014.986169

Sardenberg CMB. Liberal vs. Liberating Empowerment: A Latin American Feminist Perspective on Conceptualizing Women's Empowerment. IDS Bulletin. 2008;39(6):18–27.

Shahabuddin ASM, Nöstlinger C, Delvaux T, Sarker M, Bardají A, Brouwere VD, et al. (2016) What Influences Adolescent Girls' Decision-Making Regarding Contraceptive Methods Use and Childbearing? A Qualitative Exploratory Study in Rangpur District, Bangladesh. PLoS ONE 11(6): e0157664. https://doi.org/10.1371/journal.pone.0157664

Shahabuddin A, Nöstlinger C, Delvaux T, Sarker M, Delamou A, Bardají A, et al. Exploring Maternal Health Care-Seeking Behavior of Married Adolescent Girls in Bangladesh: A Social-Ecological Approach. PLoS ONE. 2017. 12(1): e0169109. https://doi.org/10.1371/journal.pone.0169109

Society for Adolescent Medicine. Guidelines for Adolescent Research: A Position Paper of the Society for Adolescent Medicine. Journal of Adolescent Health. 2003; 33: 396–409.

Starre AM, Ezeh A, Barker G, et al. Accelerate progress—sexual and reproductive health and rights for all: report of the Guttmacher–Lancet Commission. Lancet. 2018; 391: 2642–92

Straus MA, Hamby SL, Boney-McCoy S, et al. The revised Conflict Tactics Scales (CTS2): Development and preliminary psychometric data. Journal of Family Issues. 1996; 17(3): 283–316. http://dx.doi.org/10.1177/019251396017003001

UNFPA. Independent country programme evaluation annexes: Bangladesh (2012-2016). 2016. New York: UNFPA. https://www.unfpa.org/sites/default/files/admin-resource/Bangladesh_CPE_Final_Report_Annexes_v2.pdf

Wamoyi J, Heise L, Meiksin R, Kyegombe N, Nyato D, Buller AM. Is transactional sex exploitative? A social norms perspective, with implications for interventions with adolescent girls and young women in Tanzania. PLoS ONE. 2019. 14(4):e0214366. https://doi.org/10.1371/journal.pone.0214366

World Health Organization. *Putting Women First: Ethical and Safety Recommendations for Research on Domestic Violence Against Women.* Geneva: World Health Organization; 2001.

Yount KM, VanderEnde KE, Dodell S, Cheong YF. Measurement of Women's Agency in Egypt: A National Validation Study. Social Indicators Research. 2016;128(3):1171-92.

Yount KM, Khan Z, Miedema S, Cheong YF, Naved RT. The Women's Agency Scale 61 (Was-61): A Comprehensive Measure of Women's Intrinsic, Instrumental, and Collective Agency. 2020. Available at SSRN: https://ssrn.com/abstract=3670180 or http://dx.doi.org/10.2139/ssrn.3670180

Yount KM, Cheong YF, Khan Z, et al. Women's participation in microfinance: Effects on Women's agency, exposure to partner violence, and mental health. Social Science & Medicine. 2021;270:113686.

Yount KM, Naved RT, et al. Development and Validation of the Economic Coercion Scale-20 (ECS-20): A Short-Form of the ECS-36. Journal of Interpersonal Violence. Submitted.

Budget

2					
3	*Grant N[illegible]*				
4	*Project Title:*	Balika bodhu: Addressing denial of sexual and			
5	*Donor Name:*	DFATD			
6	*Budget Period:*	01/01/2023-31/03/2025			
7	*Budget Code:*				
8	*PI Name and Email:*	Ruchira Tabassum Naved, PhD, ruchira@icddrb.org			
9	*Budgeted Amount:*				
10					
11			***Year 3***	***Year 4***	
12	*SL*	*Budget Headings*	*Total Year-3 (Apr'23 to Mar'24)*	Year 4(Apr'24-Mar'25)	*Total Revised Budget (Y1-Y5)*
13	1.1	***Remuneration - Local Employees***	108,674	84,224	192,898
30	*1.2*	*Fees - Subcontractors with an Arm's Length Relationship with the Organization*	28,519	47,878	76,398
34	*1.3*	***Reimbursable Costs***	25,841	26,929	52,769
35	*1.3.1*	*Travel Cost*	13,023	13,465	26,488
38	*1.3.2*	*Other Training Costs*	1,650	1,912	3,562
40	*1.3.3*	*Recipient Country Government Employees*		-	
41	*1.3.4*	*Goods, Assets and Supplies*	3,500	-	3,500
44	*1.3.5*	*Project Administration costs directly related to the Project*	7,667	11,552	19,220
63		***Total Direct costs***	163,034	159,031	322,065
64	*1.4*	***Indirect Costs/Overhead (14.38%)***	23,444	22,869	46,313
65		***Total***	186,479	181,899	368,378

Time plan and implementation

	2022			2023											
**Project activities**	Oct	Nov	Dec	Jan	Feb	Mar	Apr	May	Jun	Jul	Aug	Sep	Oct	Nov	Dec
Tools and protocol development and IRB approval	■	■	■												
Intervention development		■	■	■	■	■	■	■	■	■	■	■			
**Baseline data collection**															
Recruitment and training of data collectors												■	■		
Quantitative and qualitative data collection													■	■	■
Quantitative data cleaning														■	■
**Intervention Implementation**															■

**Timeline (conti..)**															
	2024												2025		
**Project activities**	Jan	Feb	Mar	Apr	May	Jun	Jul	Aug	Sep	Oct	Nov	Dec	Jan	Feb	Mar
Quantitative data cleaning	■														
Qualitative data transcription and coding	■	■	■												
Data analysis		■	■	■	■										
Writing report and manuscript						■	■	■	■						
Intervention Implementation	■	■	■	■	■	■	■	■	■	■	■				
**End line data collection**															
Recruitment and training of data collectors											■	■			
Endline data collection (quantitative and qualitative)												■	■	■	
Quantitative data cleaning												■	■	■	
Qualitative data transcription and coding												■	■	■	
Data analysis													■	■	■
Writing report and manuscript													■	■	■
Dissemination															■

Annex B. Gender Analysis Tool

Gender Analysis of sexual and reproductive health and rights of married adolescent girls In Bangladesh

Relation to x health problems:	Are there differences between male and female in	How do the biological differences between women and men influence their	How do the different roles and activities of women and men affect their	How do gender norms/values affect women and men's	How do access to and control over resources affect women and men's
Vulnerability: Incidence Prevalence	In general, women are more vulnerable to experience SRHR related problems compared to their male counterpart. This difference becomes larger at younger age. For example, a higher proportion of women experience intimate partner violence compared to men.	Biological determinants combined with social norms increase women's vulnerability to maternal mortality, SRHR, and intimate partner violence, more significantly for the MAGs due to their young age status. For example, only women can conceive, but they usually cannot decide when to have their first child.	Gender inequalities are derived from the typical social dispersed roles and activities imposed by the gender norms and ideology which expect women to bear the responsibilities silently, rather than taking part in the decision making, e.g., it is considered to be a girl's responsibility to rear children, whereas men are expected to earn the livelihood. Again, collecting/buying the family planning products are men's roles in our country.	Gender norms and values in many aspects may affect everyday actions and behaviours of women and girls and increase vulnerability of denial of SRHR and violence. Marrying off daughters at an early age is one of the most common gender norms in many developing countries. Parents tend to practice 'Child Marriage' focusing on the financial security and fearing the increase of dowry. One other norm is also established in the society that preventing pregnancy is women's responsibility. Moreover, women endure violence, especially sexual violence, because disclosure may result in blaming the	Since men hold the decision making power in most marital homes due to the structure of patriarchal society, women thus have limited access and control over resources regarding SRHR making them more vulnerable.

				victims and damage their reputation in the community.	
Health seeking behaviour	MAGs are lagged behind compared to their male counterparts in seeking health care services related to their SRHR.	Different biological states create disparity in health seeking behaviour between men and women, e.g., pregnant women in their last trimester are not usually feasible to seek health services alone.	Husbands and in-laws are main decision makers in the family in case of health care seeking of MAGs. This disproportionately affect the heath care seeking of MAGs compared to their male partners.	One of the established gender norms in the society include “pregnancy is normal and it does not require any extra care”. Also, as women disclose their SRHR problems rarely they seek less care for that.	In south Asian countries, women generally find themselves in subordinate positions to men, and are largely excluded from decision-making, due to their limited access to and control over resources, and are restricted in their mobility which hinders them from achieving SRHR related health care.
Ability to access health services	Due to lack of mobility and hence lack of knowledge women usually have less access to health services<br><br>(WHO, 2021)	[May not be applicable]	Because of diverse and overloaded roles in marital families, women, especially MAGs have less opportunities and limited mobility to access services related to SRHR.	Women are expected to accept the SRHR choice and decisions of husbands and their in-laws. Thus, may not consider to seek services for these issues.	As women lack access to financial resources, they often seen unable to access emergency health care services related to SRHR.
Experience with health services and health providers	Usually, women are not treated equally to men in the health services and by health care providers.	[May Not be applicable]	MAGs are commonly denied the choice and consent regarding SRH care seeking such as family planning, when to conceive, how many children to have etc. For example, the health facilities usually do not allow women to terminate pregnancy without consent of their husbands.	MAGs are expected to be submissive and expected to adapt any unfair outcomes of SRHR and in their intimate relationships. Therefore, they may face discrimination from health service providers that is such less attentions or blaming attitudes toward girls.	Limited or no access to and control over resources at this early age and having no or little voice, MAGs may face devaluating attitudes from health providers.

Preventive and treatment options, responses to treatment, prevention or rehabilitation	[May not be Applicable]	[May not be Applicable]	See row 5 column 4	See row 5 column 5	See row 5 column 6
Outcome of health problem: e.g. detection, prevention, recovery, death	Girls who marry at early age are more often report unintended pregnancy, face malnutrition during pregnancy, experience obstetric complications and maternal death, have preterm or low-weight birth, and exhibit postnatal depression.	[May not be applicable]	Specifically due to reproductive roles, MAGs may face particular sexual and reproductive and mental health problem as consequences of child marriage and early pregnancy.	Gender norms demand a girl to have child as early as possible after marriage. Marrying and bearing child early when, the girl is not ready physically and mentally, worsen the complications of pregnancy and other reproductive health outcomes.	Since girls have limited or no decision making power and access and control over resources, the physical and mental health outcome of child marriage could be worse and they may not be able to avail emergency health service needed due to their economic and resource shortage.
Consequences (economic & social, including attitudinal)	Child marriage and early pregnancy limit adolescent girl's opportunity to education and income.	[May not be applicable]	Different and dual responsibilities such as reproductive role with household regular works, an adolescent girl may face longer health and economic consequences like pregnancy complications. Different gender roles may shrink girls' opportunities to recover their economic and emotional loss as girls have less employment scope.	Gender norms could be devastating for adolescent girls in context of early pregnancy. They have lower position within family and society. On top of that due to under age they may face discrimination and risk of domestic violence.	Lack of resource may hinder girls from accessing economic opportunities and social networks in long run.
